# *N*-Pyrazinoyl Substituted Amino Acids as Potential Antimycobacterial Agents—the Synthesis and Biological Evaluation of Enantiomers

**DOI:** 10.3390/molecules25071518

**Published:** 2020-03-27

**Authors:** Martin Juhás, Lucie Kučerová, Ondřej Horáček, Ondřej Janďourek, Vladimír Kubíček, Klára Konečná, Radim Kučera, Pavel Bárta, Jiří Janoušek, Pavla Paterová, Jiří Kuneš, Martin Doležal, Jan Zitko

**Affiliations:** 1 Charles University, Faculty of Pharmacy in Hradec Králové, Akademika Heyrovského 1203, Hradec Králové, Czech Republic; kuceroval@faf.cuni.cz (L.K.); horaceko@faf.cuni.cz (O.H.); JANDO6AA@faf.cuni.cz (O.J.); kubicek@faf.cuni.cz (V.K.); konecna@faf.cuni.cz (K.K.); kucerar@faf.cuni.cz (R.K.); bartp7aa@faf.cuni.cz (P.B.); janousj2@faf.cuni.cz (J.J.); kunes@faf.cuni.cz (J.K.); dolezalm@faf.cuni.cz (M.D.); 2 University Hospital Hradec Králové, Department of Clinical Microbiology, Sokolská 581, 500 05 Hradec Králové, Czech Republic; pavla.paterova@fnhk.cz

**Keywords:** amino acids, antibacterial, antimycobacterial, cytotoxicity, pyrazinamide, tuberculosis

## Abstract

Tuberculosis is an infectious disease caused by *Mycobacterium tuberculosis* (Mtb), each year causing millions of deaths. In this article, we present the synthesis and biological evaluations of new potential antimycobacterial compounds containing a fragment of the first-line antitubercular drug pyrazinamide (PZA), coupled with methyl or ethyl esters of selected amino acids. The antimicrobial activity was evaluated on a variety of (myco)bacterial strains, including Mtb H37Ra, *M. smegmatis, M. aurum, Staphylococcus aureus, Pseudomonas aeruginosa*, and fungal strains, including *Candida albicans* and *Aspergillus flavus*. Emphasis was placed on the comparison of enantiomer activities. None of the synthesized compounds showed any significant activity against fungal strains, and their antibacterial activities were also low, the best minimum inhibitory concentration (MIC) value was 31.25 µM. However, several compounds presented high activity against Mtb. Overall, higher activity was seen in derivatives containing l-amino acids. Similarly, the activity seems tied to the more lipophilic compounds. The most active derivative contained phenylglycine moiety (PC-d/l-Pgl-Me, MIC < 1.95 µg/mL). All active compounds possessed low cytotoxicity and good selectivity towards Mtb. To the best of our knowledge, this is the first study comparing the activities of the d- and l-amino acid derivatives of pyrazinamide as potential antimycobacterial compounds.

## 1. Introduction

Tuberculosis (TB) is a highly contagious infection caused by *Mycobacterium tuberculosis* (Mtb) and was responsible for over 1.5 million deaths worldwide in 2018, which ranks it in the top 10 causes of deaths and the leading cause of dying due to a single infectious agent according to the World Health Organization (WHO) [1]. Most often, TB affects the lungs of the infected individuals, although the cases of so called extra-pulmonary TB are well documented as well. Despite great advances and many clinically successful drugs, a desired cure still eludes the reaches of modern medicine [1].

The treatment of TB, as well as other bacterial infections nowadays, is hindered by growing drug resistance (DR). For TB, the WHO differentiate multidrug-resistant tuberculosis (MDR-TB) and extensively drug-resistant tuberculosis (XDR-TB). Infections with MDR or XDR strains constitute an enormous financial burden for health-care systems (≥6000 $ per person for DR TB vs. 1000 $ per person for non-DR TB) and pose significant therapeutic problems compared to non-DR TB. The most recent global data reports the success rate for the treatment of MDR-TB at only 56%, for XDR-TB lowered down to 36%, in comparison with >85% for non-DR TB [1]. The drug-resistant cases require the use of more toxic second-line antituberculars, often in further combinations with other drugs. In some cases, even this regimen is insufficient. Thus, the search for new antimycobacterial agents and improvements of the current ones is still a standing research area.

Mtb is not the only mycobacterium responsible for severe infections. The importance of other so called nontuberculous or atypical mycobacteria (NTM) increases rapidly [2]. Nowadays, the most important NTMs are slow-growing *M. avium complex* and *M. kansasii* and rapid-growing *M. abscessus* and *M. fortuitum*, with many others following [3]. The overall colonization of NTM is enormous. They likely reside in soil, pipes and water supplies, causing problems, e.g., in hospital care. As opposed to Mtb, the NTM infections do not require reporting to the authorities. Therefore, the incidence of infections caused solely by NTM is difficult to estimate. Infections involving NTM are often difficult to handle, employing a combination therapy of standard antituberculars and other antibacterial drugs [4].

Pyrazinamide (PZA) has been used for the treatment of TB since 1952 [5]. So far, several potential mechanisms of action (MoA) have been proposed [6]; however, none seem to be the definite one. Firstly, PZA is activated (hydrolysed) by the enzyme pyrazinamidase to pyrazine-2-carboxylic acid (pyrazinoic acid, POA), which then acts on several targets [7]. Previously, the activity of PZA (after hydrolysis to POA) was attributed only to non-specific effects linked to the acidification of cytoplasm and the disruption of the membrane potentials [8]. However, nowadays several specific enzymatic targets are being studied. Notable is the inhibition of fatty acid synthase I (FAS-I) [9,10], the disruption of trans-translation via interaction with ribosomal protein S1 (RpsA) [11], the inhibition of aspartate decarboxylase (PanD) [12], and the inhibition of quinolinic acid phosphoribosyltransferase (QAPRTase) [13]. Several other proteins have been suggested as targets of PZA/POA [14,15,16,17]; however, PanD has recently been suggested by some authors as the likely prevalent target [18].

PZA represents one of the most important drugs in modern TB treatment. Its biggest advantage over other antitubercular drugs is in vivo efficiency (after metabolization) in penetrating into lung granulomas and thus its ability to act on dormant mycobacteria [19]. This effect is lacking in most of the first-line antituberculars.

In search for enhancing the activity of PZA, several derivatives were prepared [20]. Most promising results were noted for the ester prodrugs of POA [21]. The inhibition of Mtb growth exerted by POA esters in vitro, also observed in PZA-resistant strains, was considerable [22]. Nevertheless, only sterically protected or long carbon chain esters were stable enough in plasma and could thus preserve their antitubercular activity in vivo [23,24]. As a solution, *N*-alkyl carboxamide derivatives of PZA were proposed. These proved to be significantly more stable in plasma and mycobacterial homogenate, although they were consequently inactive against Mtb even in vitro [24]. Our research group had also contributed towards the preparation of many active compounds containing the PZA core, focusing mainly on *N*-aryl substituted compounds presenting micromolar activities in vitro [25,26]. For more information about the current state of the pyrazinamide-derivatives research, the reader is encouraged to read the recent review by Correa and Fernandes [20].

**Figure 1:**
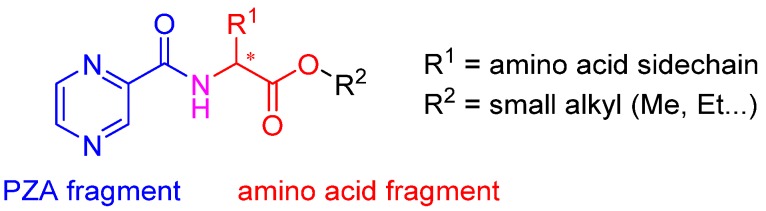
General structure of the proposed compounds. Asterisk symbol (*) denotes position of the stereo center.

Derivatives combining the PZA core with amino acid (aa), as presented in Figure 1, have caught very little attention so far, despite the fact that amino acid derivatization is very common in antimicrobial research [27,28,29]. The reason for this is probably the undesired high stability seen in *N*-alkyl derivatives of PZA [24]. Up to this point, the only data on the aa derivatives of PZA and their antimycobacterial activity are those found in older research by Kushner et al. [30], Badie and Azab [31], and Pinheiro et al. [32]. In a very recent study by Panda et al. [33], more derivatives of structure presented in Figure 1 were tested on several (myco)bacterial strains showing mild activity at neutral pH. Nevertheless, molecules of the mentioned structure have been tested for their other applications, e.g., as antihistamines [34], insecticides, anthelmintics [35], or proteasome inhibitors [36].

As a potential drug, PZA core substituted with amino acid (aa) may present numerous advantages in comparison with simple aliphatic or aromatic amides and esters. Compared to amides, small PZA-derived peptides could be more easily handled by Mtb peptidases due to their natural similarity to the peptides (compared to amides) while being stable enough in plasma medium to reach the site of infection (compared to esters). As for any other amidic-type drugs, the hydrolysis by host peptidases (amidases) diminishing the activity can occur. However, at this stage of the drug design, this issue, although important, is very troublesome to address without relevant experimental data concerning the exact MoA and the plasma stability of the proposed compounds. In aa prodrugs of antimicrobial drug dapson [29], the half-life in human plasma was shown to be in order of tens to hundreds of minutes, based on the used aa. If such stability was also observed for our title compounds, it would rank them—at least from the stability perspective—among aliphatic long carbon chain esters of POA (half-life in order of tens of minutes [24]) which are stable and active in macrophage model of Mtb infection. Similarly, they would be hydrolysed considerably quicker than aliphatic amides (half-life in order of thousands of minutes [24]). Therefore, we believe that the proposed derivatives could act as both efficient and sufficiently stable prodrugs of POA but at the same time we do not rule out the possibility that the presented derivatives can be active in their amidic, non-hydrolysed form.

To test these hypotheses, we have decided to synthesize a series of *N*-pyrazinoyl substituted amino acid derivatives and test their activity against a range of mycobacterial, bacterial, and fungal strains available to our research group. Furthermore, to our knowledge, we are the first to investigate the differences in the biological activities of compounds derived from l- and d-aa in both mildly acidic (pH 6) and commonly used pH 6.6.

## 2. Results and Discussion

### 2.1. Chemistry

Thirty-nine derivatives of PZA coupled with pure l- or d-aa or with aa enantiomeric mixtures were synthesized (for structure, see Table 1, for SMILES, calculated molecular descriptors, and selected biological data, see the Supplementary Material). In compound codes, dl signifies racemic mixtures and d/l denotes the mixture of enantiomers. Several coupling strategies were evaluated concurrently. Herein, the unsuccessful strategies (Procedures 1–4) are describe only in brief outlines.

Procedure 1: Reacting pyrazinoyl chloride, (prepared as previously described in Zitko et al. [26]), with free aa and their further esterification was found unsuitable. Multiple products (not identified) were observed, as indicated by thin-layer chromatography (TLC), and the desired product was very difficult to separate. Furthermore, due to the very low solubility of aa in organic solvents, water solutions had to be employed, resulting in the undesirable hydrolysis of the pyrazinoyl chloride, further diminishing the yield.

Procedure 2: Reacting pyrazinoyl chloride with aa esters (or their hydrochlorides) partially solved the problems with solubility in organic solvents, although, side-products remained a problem.

Procedure 3: The aminolysis of methyl pyrazinoate with aa ester (hydrochlorides) in ethanol did not work, even when heated with addition of catalytic amount of 4-(dimethylamino)pyridine (DMAP).

Procedure 4: The use of standard coupling reagents as 1,1′ -carbonyldiimidazole (CDI) in anhydrous tetrahydrofurane (THF) with aa showed good results, although in some cases starting pyrazinoic acid was still detected by TLC as a small impurity difficult to separate.

**Figure 2:**
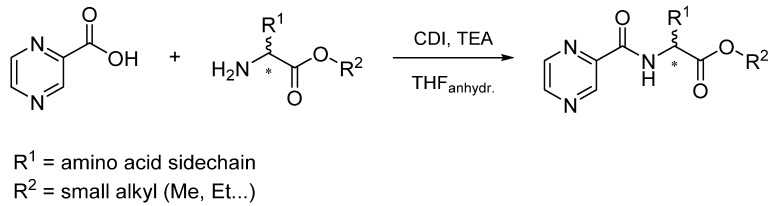
Schematic representation of Procedure 5. Asterisk symbol (*) denotes position of the stereo center. TEA—triethylamine.

Procedure 5: At last, general procedure, as described in Section 3.1.1. (and shown in Figure 2), was achieved by adjusting Procedure 4. The method employs esters of aa instead of aa with the free carboxylic group. The increased solubility of the reactants and products in used organic solvents (THF) greatly simplified the syntheses and subsequent purifications. The products were created in good yields (positively related to their lipophilicity) and without epimerization.

Based on the extensive knowledge on mycobacterial esterases [37], the use of esters over free acids was also favoured to potentially increase the bioavailability of our mostly polar title compounds, which proved to be a correct assumption based on the activities discussed further below.

We tried to synthesize Pro and His ester derivatives. However, these proved to be readily soluble in water even as esters. Therefore, we were unable to sufficiently purify them for the biological screening. PC-MeAcr derivative was obtained as a by-product by the elimination of the hydroxyl of serine methyl ester, as previously described by Pinheiro et al. [32].

All compounds were characterized by ^1^H- and ^13^C-NMR spectroscopy in hexadeuterodimethyl sulfoxide (DMSO-*d*_6_), IR spectroscopy using the attenuated total reflectance (ATR) method, and mass spectrometry. All analytical results were in agreements with the presumed structures.

In ^1^H-NMR, the amidic hydrogen was always observed as a clearly distinguishable doublet, usually around 8.0–9.5 ppm, and the usual *J* coupling constant ranged between 8.4–8.5 Hz. Pyrazine hydrogens were observed as two doublets and one multiplet at 9.1–9.4, 8.8–9.1, and 8.6–8.9 ppm, respectively. C-α hydrogen was observed as a distinguishable multiplet at 4.4–4.6 ppm in all chiral compounds. The acidic hydrogen in free carboxylic moiety was sometimes not observed due to rapid exchange with solvent, which is common in DMSO-*d*_6_^1^H-NMR. Alcoholic OH hydrogen in Ser ester derivatives was observed as a clear doublet of doublets or a triplet at 5.3 ppm. In the free acid Ser derivative, as well as in Thr esters, the OH hydrogen was sometimes indistinguishable. The representative spectra of free aliphatic aa derivative (PC-l-Ala), ester derivative (PC-l-Val-Me), and aromatic and phenylglycine (Pgl) aa ester derivatives (PC-d/l-Pgl-Me, PC-dl-Phe-Et) are enclosed in the Supplementary Material.

In the IR spectra of all the title compounds, the amidic N-H stretching was observed as a distinguishable absorption band at 3200–3300 cm^−1^, amidic C=O stretching was observed at 1640–1670 cm^−1^, and ester C=O stretching at 1730–1750 cm^−1^. Free acids presented characteristically broad O-H stretches around 3000 cm^−1^. In Ser and Thr derivatives, an aliphatic O-H stretch was visible above 3300 cm^−1^.

Amino acid esterification [38] and other common peptide synthesis procedures (e.g., *tert*-butyl group cleavage) [39,40] used to obtain the title compounds are considered enantioselective. Acidic hydrolysis was previously described to potentially cause epimerization or even racemization of aa derivatives [41]. CDI-mediated coupling in THF had been shown to cause a low degree of epimerization (5%) on one model peptidic compound [42]. Generally, no epimerization occurred in our conditions. Epimerization was seen only in the case of Pgl derivative (PC-d/l-Pgl-Me) synthesized using the d-Pgl methyl ester hydrochloride. The composition of the mixture of enantiomers was further investigated by optical rotation measurements and chiral HPLC. Comparing the literature value of the specific rotation of l-enantiomer ([α]_D_ = +88.8° [43]) with the measured value of our sample [α]_D_ = −47.3°, it is clear that d-enantiomer dominates the mixture of enantiomers as judged from the sense of rotation. According to the chiral HPLC, the sample consists of two enantiomers represented by peaks in a ratio of approx. 2.1:1, based on the area under the curve (AUC). We thus conclude that the compound PC-d/l-Pgl-Me is a mixture of d- and l-enantiomer in ratio of 2.1:1 in favour for d-enantiomer. The epimerization of Pgl is known to occur very easily using standard coupling conditions and thus stereoselective synthesis would require specific reagents not employed in this work [44]. Chiral HPLC analysis was performed for enantiomeric mixture PC-d/l-Pgl-Me, racemic mixtures (PC-dl-Ala-Me, PC-dl-Leu-Et, PC-dl-Phe-Et, PC-dl-Val-Et) and for the samples of which we had the complete couple of antipodes. The chromatograms are presented in the Supplementary Material.

### 2.2. Biological Evaluation

#### 2.2.1. Antimycobacterial Activity Screening

Mycobacterial screening of the synthesized compounds was done using microdilution assay (Microplate Alamar Blue Assay, MABA [45]) on *M. smegmatis* DSM 43465 (ATCC 607), *Mycobacterium aurum* DSM 43999 (ATCC 23366), and the avirulent strain of Mtb H37Ra ITM-M006710 (ATCC 9431)—for results, see Table 2 and Table 3. The minimum inhibitory concentration (MIC) values were measure in µg/mL. To account for molecular weight, the inhibitory activity (expressed as MIC) of the discussed compounds were recalculated to µM concentration. Fast-growing *M. smegmatis* and *M. aurum* are used as surrogate model organisms in antimycobacterial research [46,47]. Mtb H37Ra is commonly used as an accessible valid alternative for the virulent strain [48]. The selected title compounds were also tested in pre-screening against a virulent strain of Mtb H37Rv CNCTC My 331/88 (ATCC 27294), *M. kansasii* CNCTC My 235/80 (ATCC 12478), and *M. avium* ssp. *avium* CNCTC My 80/72 (ATCC 15769). The activity of PC-MeAcr was in accordance with previously published results (MIC on Mtb H37Rv was 50 µg/mL in our study as well as in the study by Pinheiro et al. [32]). Although PC-MeAcr might have been considered cytotoxic due to the acrylate group, no significant cytotoxicity was noted on the human HepG2 cell line, as presented in Table 4. For the full results of this pre-screening, see Table S2 in the Supplementary Material.

For the purposes of this article, only compounds with MIC values lower than 31.25 µg/mL (roughly corresponding to 100 µM based on molecular weight) were considered as active.

Notable activity was detected only against Mtb H37Ra at pH 6. No significant activity was detected against *M. smegmatis* at neither pH 6 nor pH 6.6, low activity was noted only for PC-MeAcr (MIC = 31.25 µg/mL, pH 6.6). Only one compound (PC-d/l-Pgl-Me) showed notable activity against *M. aurum* (MIC = 31.25 µg/mL, pH 6). If not stated otherwise, the following discussion concerns activity against Mtb H37Ra at pH 6.

Limited data on similar aa derivatives of PZA are available in the literature. In the work of Panda et al. [33], the l-derivatives of non-esterified aa (Leu, Val, allo-Ile, Met, Phe, Trp) were tested. These compounds were more or similarly active at pH 7 compared to isoniazid (INH) (> 70% growth inhibition at 30 µg/mL). However, as a different Mtb strain and methodology was used in the study of Panda et al. [33], direct comparison to our study is not applicable. PC-Asp-diEt and some atypical aa derivatives of the structure presented in Figure 1 were tested previously by Kushner et al. [30] with no activity, which is in accordance with our results presented in Table 2 and Table 3.

##### pH Dependence

Based on one of the proposed MoA of our compounds, that is POA-releasing prodrugs, all derivatives were tested at mildly acidic pH 6 as required for the in vitro activity of PZA/POA, and the results were compared to the activities at the commonly used pH 6.6 [22]. The specific pH value for the acidic measurement was selected to balance the activity of tested compounds with the growth rate of cultivated mycobacteria [49]; furthermore, a similar pH is also found in macrophages, where the intracellular pathogen of Mtb resides [50], making these conditions closer to reality. The necessity of acidic conditions for the in vitro activity of PZA and POA is a well-known paradigm. A similar pH-activity dependence is known also for POA esters [22]. However, Pinheiro et al. [32] and den Hertog et al. [51] have shown that the activity of PZA and POA may also be observed under non-acidic conditions. According to den Hertog et al. [51], the in vitro activity of PZA is probably tied to specific metabolic and energetic effects occurring in acidic medium, although they concluded that the same effects could also be induced by other stressors, e.g., low temperature or starvation. In general, these implications do not affect the correctness of the in vitro measurements presented in this manuscript or elsewhere but rather propose new ways to investigate the MoA of PZA.

Based on the results presented in Table 2 and Table 3, the impact of the pH value on the activity of our compounds against Mtb was substantial. At least an 8-fold decrease in MIC was observed for all active compounds, corresponding to the change from hundreds of µg/mL to tens of µg/mL (or lower). The effect of pH on the activity against other mycobacterial strains was less profound, similar to PZA itself, which could be suggesting PZA-linked MoA.

##### Lipophilicity and Sidechain Effects

**Figure 3:**
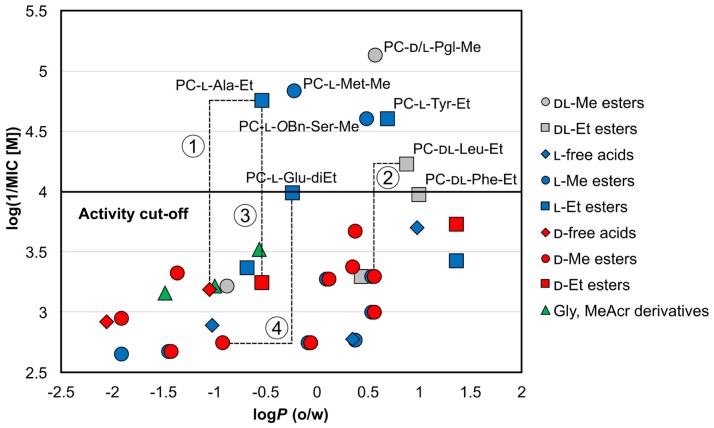
Scatter plot representing antimycobacterial activity and calculated lipophilicity of the compounds. The colours denote stereochemistry; the shape of the symbols distinguishes between esters and free acids. Gly and MeAcr derivatives are presented as green triangles. The MIC values of the majority of the derivatives in the non-active region were not determined precisely (see Table 3).

Mycobacterial membrane is very lipophilic. Compared to other bacteria, it contains unusually high concentrations of specific lipidic molecules, e.g., mycolic acids [52]. Consequently, highly lipophilic low molecular weight compounds are expected to penetrate the membrane through a simple passive diffusion more easily. Nevertheless, the exact physico-chemical requirements relevant to the Mtb membrane are still unknown [53]. To assess the impact of the lipophilicity of the synthesized compounds on their activities, we investigated the relationship between calculated log*P* and respective MIC values at pH 6 (as no significant activity was recorded pH 6.6). All compounds were categorized considering their chemical nature (free carboxylic acid/ester) and enantiomer aspects (as discussed later). The log*P* (o/w) chemical descriptor, hereafter referred to as log*P*, was calculated by Molecular Operating Environment (MOE) 2019.0101 (Chemical Computing Group (CCG), Quebec, Canada) [54]. The resulting scatter plot of log*P* and antimycobacterial activity presented as log(1/MIC [M]) (a higher value means better activity) is shown in Figure 3. The calculated log*P* and MIC (µM) values at pH 6 are contained in Table 3.

Clearly, for the same aa derivatives, the least active compounds in categories free acid–methyl ester–ethyl ester are embodied by derivatives with free carboxylic moiety (represented by gems in Figure 3). Furthermore, ethyl esters (represented by squares) were generally more active than methyl esters in our study. This general observation can be attributed to their higher lipophilicity (as indicated by increased log*P* in Table 3) compared to the free acids. As an illustrative example, in l-Ala derivatives, the MIC value of the ethyl ester is at least 64-times lower compared to that of the free acid (PC-l-Ala-Et MIC = 3.91 µg/mL vs. PC-l-Ala MIC ≥ 250 µg/mL), with the log*P* difference of approximately 0.5 (−0.53 for ethyl ester vs. −1.02 for free acid)—line 1 in Figure 3. In Leu and Glu esters, high activity was noted only for more lipophilic ethyl esters, while the less lipophilic methyl esters were inactive (PC-dl-Leu-Et vs. PC-d-Leu-Me—line 2 and PC-l-Glu-diEt). In fact, the majority of methyl esters (represented by circles) are located below the activity cut-off in Figure 3, with the exception of highly lipophilic compounds, e.g., PC-l-*O*Bn-Ser-Me, PC-d/l-Pgl-Me. The effect of growing activity with the lengthening of the ester carbon chain had previously also been observed in derivatives of POA [21,23] or other antimycobacterial compounds [55] and it has been attributed to the above-mentioned better penetration inside the mycobacteria. However, as discussed later, other effects—as stereochemistry—seem to play an important role in structure–activity relationships in our compounds. Therefore, the effect of ester moiety on activity stays inconclusive.

When comparing the activities of our title compounds, higher activity is seen mostly in derivatives of aa with hydrophobic aliphatic or aromatic side chains.

In a homologous series of Gly, Ala, Val, Leu, and Ile derivatives, high activity was noted only for derivatives of Ala and Leu. Interestingly, no activity was seen in the similar lipophilic ester of Val or in the derivatives of mutual chain isomers Leu and Ile. The highest activity in aliphatic aa derivatives was seen in Met derivative PC-l-Met-Me, having comparable activity with PC-l-Ala-Et (MIC = 3.91 µg/mL). The activity of tested polar aliphatic aa derivatives (Cys, Ser, Thr) seemed tied mainly to the chemically protected form (active PC-l-*O*Bn-Ser-Me vs. inactive PC-l-Ser-Me with unsubstituted hydroxyl), supposedly due to a higher lipophilicity of the protected alternative.

High activities were noted for the majority of aromatic aa derivatives. The highest activity of all tested compounds was seen in PC-d/l-Pgl-Me (MIC < 1.95 µg/mL), and derivatives of Tyr were active as well. All observed results may be tied to the generally higher lipophilicity of aliphatic and aromatic compounds; however, enzymatic preferences towards the hydrolysis of specific amidic bonds may also play a significant role, as discussed later.

##### Stereochemical Aspects of Activity

Enzymatic substrate specificity is the cornerstone of catalysis, and enzymes with one-enantiomer specificity are known in microorganisms [56,57]. We were therefore curious to investigate the differences in activities of the l- and d-enantiomers of the title compounds, as they may suggest either a specific MoA of the unhydrolyzed amide, or a more facile hydrolysis of one enantiomer.

As may be seen from Figure 3, d-enantiomers (shown in red) were in general inactive. However, as discussed above, caution is needed when comparing enantiomers, as other factors, such as the overall lipophilicity, may also influence the activity. Therefore, it would be safe to compare the activities of the same esters, while comparing activity of different esters of the same aa derivative should be judicious.

Most illustrative examples following the above-mentioned rule (d-isomers are inactive) are once again represented by derivatives of Ala. A comparison of PC-l-Ala-Et with PC-d-Ala-Et (line 3) clearly shows the preference of the l-enantiomer (MIC = 3.91 µg/mL vs. ≥ 125 µg/mL, respectively). Other l-derivatives being more active were noted as well, although the activities might be biased by the ester differences, as in the case of PC-l-Glu-diEt vs. PC-d-Glu-diMe (MIC = 31.25 µg/mL vs. MIC = ≥ 500 µg/mL, line 4) or PC-l-Tyr-Et vs. PC-d-Tyr-Me (MIC = 7.81 µg/mL vs. MIC = 125 µg/mL). The activity of racemic or enantiomeric mixtures (painted in grey), as for PC-d/l-Pgl-Me and PC-dl-Leu-Et, is not possible to attribute to the exact enantiomer; although, based on the overall trends, we propose l-Leu derivative to be more active. As Pgl is not a natural amino acid, the activity could by tied to both enantiomers. We were unable to test this hypothesis as the pure l- or d-enantiomers of Pgl derivative were not synthesized. Finally, both l- and d-Trp derivatives showed similar, although very low activity against Mtb H37Ra (one or two dilution steps below the cut-off value). However, based on the observed cytotoxicity of PC-l-Trp-Et (l-enantiomer of Trp) on HepG2 cell lines (IC_50_ = 146.6 µM, Table 4), and the unchanged MIC value depending on the pH, we attributed the antimycobacterial activity of both Trp derivatives to their non-specific toxicity of unknown nature.

The only case of d-enantiomer being more active than l-enantiomer (in the case of the Pgl derivative, we are unable to distinguish the active and inactive enantiomer) was in *O*-*tert*-butyl-protected derivatives of Thr (PC-d-*Ot*Bu-Thr-Me, MIC = 62.5 µg/mL vs. PC-l-*Ot*Bu-Thr-Me, MIC ≥ 500). The activity was low, however. The reason for this preference is unknown. Nevertheless, ether-protected derivatives are in fact non-natural aa. Observed activity is still very interesting, as we noted that in bacteria the same d-enantiomer showed no activity while the l-enantiomer was active against *Staphylococcus aureus* strains (discussed further in Section 2.2.2.).

#### 2.2.2. Antibacterial Activity Screening

All compounds were tested by the microdilution broth method [58] against *Staphylococcus aureus* subsp. *aureus* CCM 4223 (ATCC 29213), *Staphylococcus aureus* subsp. *aureus* methicillin-resistant (MRSA) CCM 4750 (ATCC 43300), *Staphylococcus epidermidis* CCM 4418 (ATCC 12228), *Enterococcus faecalis* CCM 4224 (ATCC 29212), *Escherichia coli* CCM 3954 (ATCC 25922), *Klebsiella pneumoniae* CCM 4415 (ATCC 10031), *Acinetobacter baumannii* DSM 30007, ATCC 19606, and *Pseudomonas aeruginosa* CCM 3955 (ATCC 27853). The majority of the compounds did not show any activity against bacteria under testing conditions (the highest concentrations tested were 500 µM for the majority of compounds, 250 µM for PC-l-*S*Bn-Cys, and 125 µM for PC-l-*O*Bn-Ser-Me. Three compounds (PC-d-Asp-diMe, PC-d-Ile-Me and PC-l-Ile-Me) were insoluble in the cultivation medium and therefore were not tested. PC-l-Ala-Et derivative had low activity against some G- bacteria (MIC = 125 µM, see Supplementary Material), while the d-enantiomer and dl-racemate were both inactive. The PC-l-*Ot*Bu-Thr-Me derivative was active against *S. aureus*, including MRSA with MIC = 31.25 µM, suggesting that the MoA of the compound is different from the beta-lactams. No differences in MIC values read after 24 h and 48 h of incubation were noted.

In previous studies, the activities of the methyl esters of PC-Gly, PC-l-Ala, PC-l-Ser, and PC-l-Tyr against some bacterial strains were evaluated, yet no activity was noted [31], in agreement with the observations in this article. The activities of some free acid compounds reported in the study of Panda et al. [33] could be tied to the methodology differences and strain susceptibility, as no activity was seen when corresponding esters were used in our studies. For the full results (Table S4) and methodology, see the Supplementary Material.

#### 2.2.3. Antifungal Activity Screening

All compounds were tested by the microdilution broth method [59,60] against *Candida albicans* CCM 8320 (ATCC 24433), *Candida krusei* CCM 8271 (ATCC 6258), *Candida parapsilosis* CCM 8260 (ATCC 22019), *Candida tropicalis* CCM 8264 (ATCC 750), *Aspergillus fumigatus* ATCC 204305, *Aspergillus flavus* CCM 8363, *Lichtheimia corymbifera* CCM 8077, and *Trichophyton interdigitale* CCM 8377 (ATCC 9533). No compound showed any significant activity against tested fungal strains up to the highest tested concentration, which was 500 µM for the majority of compounds, 250 µM for PC-l-*S*Bn-Cys, and 125 µM for PC-l-*O*Bn-Ser-Me. Three compounds (PC-d-Asp-diMe, PC-d-Ile-Me and PC-l-Ile-Me; the same as for antibacterial testing) were insoluble in the medium and therefore were not tested. No differences in MIC values read after 24 and 48 h (72 h) of incubation were noted. For the full results (Table S6) and methodology, see the Supplementary Material.

#### 2.2.4. Cytotoxicity Screening Results

The cytotoxicity of selected active compounds was measured using the standard human hepatocellular carcinoma cell line HepG2. The used CellTiter 96 assay is based on the reduction of tetrazolium dye MTS in living cells to formazan, which is then determined colorimetrically. The reduction of the reagent is related to availability of NADH and NADPH. The decline in levels of these metabolically important compounds in the cell leads to the decreased production of formazan.

The parameter IC_50_ was used as a measure of cytotoxicity, which allows for the quantitative comparison of the toxicity among tested compounds (Table 4). Overall, the toxicity of the compounds was very low, some compounds had the IC_50_ values above the tested range (PC-l-Ala-Et). The highest cytotoxicity illustrated by its lowest IC_50_ value was noted for the aromatic compound PC-l-Trp-Et (IC_50_ = 146.6 µM).

The selectivity index (SI) for Mtb was calculated for the most active compounds according to the equation *SI* = *IC*_50_(*µM*)/*MIC*(*µM*), as used by Bagla et al. [61], and the results are presented in Table 4. The tested compounds were more active against Mtb (SI > 1) than their cytotoxic effect against human cells, represented by the HepG2 cell line cytotoxicity experiment. Two of the most active compounds (PC-l-Ala-Et and PC-d/l-Pgl-Me) had SI > 40, which suggests a good cytotoxicity profile for potential further development.

## 3. Materials and Methods

### 3.1. Chemistry

All chemicals were of reagent or higher grade of purity. l- and d-aa were purchased from Fluorochem (Derbyshire, UK), TCI (Tokyo, Japan), Alfa Aesar (Kendal, Germany) or Sigma-Aldrich (Steinheim, Germany). The 1,1′ -carbonyldiimidazole (CDI) reagent was bought from Sigma-Aldrich, the solvents were from Sigma-Aldrich or Penta (Prague, Czech Republic). Thin-layer chromatography (TLC) was performed on Silica 60 F_254_ TLC plates (Merck, Darmstadt, Germany).

#### 3.1.1. General Procedure of Coupling Reaction

To a round bottom flask, 248 mg (2 mmol) of pyrazinoic acid (in the codes of final compounds denoted as PC) was suspended in anhydrous THF (3 mL), 324 mg (2 mmol) of CDI was added and the mixture was heated to 60 °C and stirred for 1 h. Afterwards, the flask was cooled down to laboratory temperature and 2 mmol of amino acid or amino acid ester (mostly in hydrochloride form) was added to the mixture with 300 µL (2.14 mmol) of triethylamine. After the addition, the mixture reacted for 2 h at laboratory temperature and was evaporated on a rotary evaporator. The solid residue was suspended in water (10 mL) and extracted to ethyl acetate (EtOAc) using repetitive extractions (typically twice with 15 mL) in separatory funnel. After each separation, the presence of the product in the phases was monitored on a TLC (silica, methanol-EtOAc 1:1). Finally, the organic layers were combined and washed with 10 mL of 0.01 M HCl and 10 mL of saturated NaCl solution. At the end, the organic phases were mixed with approximately 50 mg of activated carbon, stirred for 10 min, filtered and evaporated to dryness. The final product was left in the desiccator overnight and purified further using flash chromatography if necessary.

#### 3.1.2. General Procedure for Esterification of Amino Acids

Methyl esters of d-Ile and *O*-benzyl-Ser derivatives were prepared as described in Li and Sha [38] using 2 mmol of amino acid, 0.5 mL of trimethylsilyl chloride (TMSCl), and 10 mL of methanol. The reaction mixture was evaporated to dryness and stored in a desiccator.

#### 3.1.3. Deprotection of *O*-*Tert*-Butyl-Protected Hydroxy Amino Acids

Deprotection followed a common deprotection protocol. A total of 0.5 mmol of *O*-tert-butyl-protected derivative was suspended in 1 mL of deprotection solution (0.95 mL trifluoracetic acid, 0.025 mL triisopropyl silane, 0.025 mL distilled water) and stirred for 1 h. Subsequently, the mixture was evaporated to dryness and the product was purified using flash chromatography.

#### 3.1.4. General Procedure for Hydrolysis of Esters

The preparation of l-Ala derivative was achieved by acidic hydrolysis of the parent ester. A total of 0.5 mmol (approx. 100 mg) of ester was heated in 10 mL of 0.02 M solution of hydrochloric acid at 45 °C until the spot of the ester disappeared on the TLC plate. Then, the mixture was left to evaporate, and crude product was recrystallized from water.

#### 3.1.5. Flash Chromatography

The compounds were adsorbed on silica gel and purified on a PuriFlash XS420 chromatographic system (Interchim, Montluçon, France) using a gradient elution. We used a combination of silica gel and DIOL-silica gel columns (Interchim) to achieve better separation of the compounds. As the mobile phase, a mixture of hexane and EtOAc was used.

#### 3.1.6. HPLC Analysis and Optical Rotation Measurements

The chemical and enantiomeric purity of the compounds were tested on a Shimadzu chromatograph composed of a DGU-20A3 degasser, an LC-20AD binary gradient pump, an SIL-20AC autosampler with a 100-μL sample loop, a CTO-20AC thermostated column oven, and an SPD-M20A photodiode array detector (UV-PDA) controlled by a CBM-20A system controller from computer software LabSolutions (Shimadzu, Kyoto, Japan). The chiral separation was done on a Lux Amylose-1 (amylose tris(3,5-dimethylphenyl)carbamate) (250 × 4.6 mm) column, particle size 5 μm (Phenomenex, Germany). The mobile phase consisted of methanol or acetonitrile–50 mM aqueous NaClO_4_ or methanol–0.2% (*v*/*v*) acetic acid, in specified volumetric ratios. The exact conditions are summarized in Table S1 of the Supplementary Material. The flow rate was 1 mL/min and the injection volume was 1 μL. UV detection was performed in single wavelength mode (270 nm). For the collection of the UV spectra, a photodiode array scan mode (in the range 190–400 nm with a 1 nm step) was used.

Optical rotation measurements were used for the investigation of PC-d/l-Pgl-Me derivative and for further investigations of compounds without exact antipodes (different esters of d- and l-aa). Polarimeter Krüss P3000 (A. Krüss Optronic GmbH, Hamburg, Germany) was used. Compounds PC-d/l-Pgl-Me and PC-l-Met-Me were dissolved in chloroform (to compare the obtained specific rotation with literature), and other compounds were dissolved in methanol to final concentrations c = 5 to 20 mg/mL (based on observed rotation and/or available amount of samples). The optical rotation was measured in a 5 cm long glass cuvette using a 589 nm wavelength at laboratory temperature (25 ± 1 °C). All measurements were done in triplicates and the average values are reported.

#### 3.1.7. NMR, IR Analysis, and Melting Point Measurements

The ^1^H– and ^13^C–NMR spectra of the compounds were recorded on Varian VNMR S500 (Varian, Palo Alto, CA, USA) at 500 MHz for ^1^H and 126 MHz for ^13^C. Chemical shifts were reported in ppm (δ) and were referred indirectly to tetramethylsilane via the signal of the solvent (2.49 for ^1^H and 39.7 for ^13^C in DMSO-*d*_6_). The infrared spectra were recorded with spectrometer FT-IR Nicolet 6700 (Thermo Scientific, Waltham, MA, USA) using the attenuated total reflectance (ATR) methodology on germanium crystal. Melting points (mp) were measured on Stuart SMP30 (Bibby Scientific Limited, Staffordshire, UK) using the capillary method. For compounds gained in small amounts, mp was measured using Kofler melting point apparatus with a microscope.

#### 3.1.8. LC-MS and GC-MS Analysis

Advantage Max ion-trap LCQ mass spectrometer with ESI ion source (Thermo Finnigan, San Jose, CA, USA) was used for confirmation of the molecular weight of compounds containing free carboxylic group in negative mode (direct injection). All spectra were processed with Thermo Finnigan Xcalibur software (Thermo Finnigan, San Jose, CA, USA).

The GC-MS analysis was performed on a GCMS-QP2010 Plus system operating in EI mode at 70 eV (Shimadzu, Tokyo, Japan). The chromatographic separation was carried out on a Rtx-5MS column (30 m × 0.25 mm × 0.25 μm, Restek, USA). The column temperature was increased by 20 °C/min, from 130 to 320 °C, and this temperature was maintained for 2 min. The injector temperature was 240 °C and the carrier gas flow rate (helium) was set to 1 mL/min. The detection range was *m*/*z* 110–700, and the ion source temperature was held at 220 °C. The sample (1 µL) was introduced into injector in split mode (ratio 1:1). The data were recorded and analysed by the GCMS Solution Software ver. 4.45 (Shimadzu, Tokyo, Japan).

### 3.2. Analytical Results

All the analytical results for the synthesized compounds are presented in this section. If available, the CAS number is indicated. The results were in accordance with the presumed structure.

*PC-d-Ala ((pyrazine-2-carbonyl)-d-alanine)*

CAS 1308937-79-2. Yield: 26%. m.p. = 175–178 °C. ^13^C-NMR (126 MHz, DMSO-*d*_6_): δ 173.74, 162.70, 147.95, 144.52, 143.70, 143.61, 47.97, 17.31. ^1^H-NMR (500 MHz, DMSO-*d*_6_): δ 12.80 (bs, 1H), 9.18 (d, *J* = 1.5 Hz, 1H), 8.94 (d, *J* = 7.6 Hz, 1H), 8.89 (d, *J* = 2.5 Hz, 1H), 8.76–8.74 (m, 1H), 4.54–4.44 (m, 1H), 1.43 (d, *J* = 7.3 Hz, 3H). IR (ATR-Ge, cm^−1^): 3370 CONH N-H stretching, 1641 CONH C=O stretching, 1549 CONH C-N stretching, 2923 COOH O-H stretching, 1736 COOH C=O stretching. MS (ESI-): [M−H]^−^ = 194.

*PC-l-Ala ((pyrazine-2-carbonyl)-l-alanine)*

CAS 117904-05-9. Yellowish solid. Yield: 95%. m.p. = 170–174 °C (lit. from ethanol 170–173 °C [63]). ^13^C-NMR (126 MHz, DMSO-*d*_6_): δ 173.72, 162.69, 147.95, 144.52, 143.69, 143.62, 47.98, 17.32. ^1^H-NMR (500 MHz, DMSO-*d*_6_): δ 9.18 (d, *J* = 1.5 Hz, 1H), 8.93 (d, *J* = 7.5 Hz, 1H), 8.89 (d, *J* = 2.5 Hz, 1H), 8.75 (dd, *J* = 2.5, *J* = 1.5 Hz, 1H), 4.54–4.44 (m, 1H), 1.43 (d, *J* = 7.2 Hz, 3H). IR (ATR-Ge, cm^−1^): 3370 CONH N-H stretching, 1641 CONH C=O stretching, 1549 CONH C-N stretching, 2872 COOH O-H stretching, 1736 COOH C=O stretching. MS (ESI-): [M−H]^−^ = 194.

*PC-dl-Ala-Me (methyl (pyrazine-2-carbonyl)alaninate)*

CAS 955039-15-3. Yellowish solid. Yield: 17%. m.p. = 53–56 °C. ^13^C-NMR (126 MHz, DMSO-*d*_6_): δ 172.63, 162.94, 147.97, 144.48, 143.78, 143.62, 52.24, 48.00, 17.00. ^1^H-NMR (500 MHz, DMSO-*d*_6_): δ 9.18 (d, *J* = 1.5 Hz, 1H), 9.16 (d, *J* = 7.1 Hz, 1H), 8.90 (d, *J* = 2.5 Hz, 1H), 8.77–8.74 (m, 1H), 4.62–4.54 (m, 1H), 3.65 (s, 3H), 1.43 (d, *J* = 7.3 Hz, 3H). IR (ATR-Ge, cm^−1^): 3258 CONH N-H stretching, 1671 CONH C=O stretching, 1537 CONH C-N stretching, 1747 COOR C=O stretching, 2993, 2951 C-H stretching. MS (EI): M^+·^ = 209.

*PC-d-Ala-Et (ethyl (pyrazine-2-carbonyl)-d-alaninate)*

Oily liquid. Yield: 45%. ^13^C-NMR (126 MHz, DMSO-*d*_6_): δ 172.13, 162.94, 147.95, 144.50, 143.74, 143.61, 60.85, 48.12, 16.99, 14.20. ^1^H-NMR (500 MHz, DMSO-*d*_6_): δ 9.18 (d, *J* = 1.5 Hz, 1H), 9.11 (d, *J* = 7.4 Hz, 1H), 8.90 (d, *J* = 2.5 Hz, 1H), 8.77–8.75 (m, 1H), 4.58–4.50 (m, 1H), 4.15–4.08 (m, 2H), 1.44 (d, *J* = 7.4 Hz, 3H), 1.18 (t, *J* = 7.1 Hz, 3H). IR (ATR-Ge, cm^−1^): 3392 CONH N-H stretching, 1675 CONH C=O stretching, 1522 CONH C-N stretching, 1736 COOR C=O stretching, 2984 C-H stretching. MS (EI): M^+·^ = 223.

*PC-l-Ala-Et (ethyl (pyrazine-2-carbonyl)-l-alaninate)*

Oily yellow liquid. Yield: 32%. ^13^C-NMR (126 MHz, DMSO-*d*_6_): δ 172.14, 162.94, 147.96, 144.50, 143.75, 143.62, 60.86, 48.13, 16.99, 14.22. ^1^H-NMR (500 MHz, DMSO-*d*_6_): δ 9.19–9.17 (m, 1H), 9.12 (d, *J* = 7.3 Hz, 1H), 8.90 (d, *J* = 2.5 Hz, 1H), 8.77–8.75 (m, 1H), 4.58–4.50 (m, 1H), 4.15–4.08 (m, 2H), 1.44 (d, *J* = 7.3 Hz, 3H), 1.19 (t, *J* = 7.1 Hz, 3H). IR (ATR-Ge, cm^−1^): 3389 CONH N-H stretching, 1674 CONH C=O stretching, 1525 CONH C-N stretching, 1738 COOR C=O stretching, 2984 C-H stretching. MS (EI): M^+·^ = 223.

*PC-d-Asp-diMe (dimethyl (pyrazine-2-carbonyl)-d-aspartate)*

Oily yellow liquid. Yield: 66%. ^13^C-NMR (126 MHz, DMSO-*d*_6_): δ 171.03, 170.96, 162.94, 148.20, 144.10, 143.82, 143.71, 52.64, 51.91, 48.75, 35.46. ^1^H-NMR (500 MHz, DMSO-*d*_6_): δ 9.27 (d, *J* = 8.4 Hz, 1H), 9.20 (d, *J* = 1.5 Hz, 1H), 8.91 (d, *J* = 2.5 Hz, 1H), 8.78–8.69 (m, 1H), 5.10–4.86 (m, 1H), 3.65 (s, 3H), 3.61 (s, 3H), 3.01 (dd, *J* = 16.6 Hz, *J* = 6.4 Hz, 1H), 2.94 (dd, *J* = 16.6 Hz, *J* = 6.4 Hz, 1H). IR (ATR-Ge, cm^−1^): 3382 CONH N-H stretching, 1679 CONH C=O stretching, 1519 CONH C-N stretching, 1737 COOR C=O stretching, 2954C-H stretching. [α]_D_ = −4.0° (methanol). MS (EI): M^+·^ = 267.

*PC-l-Asp-diEt (diethyl (pyrazine-2-carbonyl)-l-aspartate)*

CAS 882740-96-7. Yellowish solid. Yield: 25%. m.p. = 54–57 °C (lit. aqueous ethanol 64–65 °C [30]). ^13^C-NMR (126 MHz, DMSO-*d*_6_): δ 170.47, 170.35, 162.88, 148.18, 144.12, 143.78, 143.69, 61.30, 60.51, 48.87, 35.72, 14.15, 14.11.

^1^H-NMR (500 MHz, DMSO-*d*_6_): δ 9.21 (d, *J* = 8.2 Hz, 1H), 9.20 (d, *J* = 1.5 Hz, 1H), 8.91 (d, *J* = 2.4 Hz, 1H), 8.79–8.74 (m, 1H), 5.01–4.90 (m, 1H), 4.16–4.00 (m, 4H), 3.03–2.86 (m, 2H), 1.16 (t, *J* = 7.1 Hz, 6H). IR (ATR-Ge, cm^−1^): 3311 CONH N-H stretching, 1660 CONH C=O stretching, 1538 CONH C-N stretching, 1732 COOR C=O stretching, 2978 C-H stretching. [α]_D_ = 12.0° (methanol). MS (EI): M^+·^ = 295.

*PC-l-SBn-Cys (S-benzyl-N-(pyrazine-2-carbonyl)-l-cysteine)*

Yellowish solid. Yield: 84%. m.p. = 84–87 °C. ^13^C-NMR (126 MHz, DMSO-*d*_6_): δ 171.77, 162.88, 148.12, 144.20, 143.72, 143.68, 138.36, 129.00, 128.54, 127.03, 51.77, 35.41, 32.31. ^1^H-NMR (500 MHz, DMSO-*d*_6_): δ 9.21 (d, *J* = 1.5 Hz, 1H), 8.99 (d, *J* = 8.2 Hz, 1H), 8.92 (d, *J* = 2.5 Hz, 1H), 8.80–8.76 (m, 1H), 7.31–7.24 (m, 4H), 7.24–7.17 (m, 1H), 4.75–4.67 (m, 1H), 3.76 (s, 2H), 3.03–2.97 (m, 2H). IR (ATR-Ge, cm^−1^): 3357 CONH N-H stretching, 1660 CONH C=O stretching, 1519 CONH C-N stretching, 1732 COOH C=O stretching, 2916 C-H stretching. [α]_D_ = −49.3° (methanol). MS (ESI-): [M−H]^−^ = 316.

*PC-d-Glu-diMe (dimethyl (pyrazine-2-carbonyl)-d-glutamate)*

Oily yellow liquid. Yield: 57%. ^13^C-NMR (126 MHz, DMSO-*d*_6_): δ 172.87, 171.74, 163.44, 148.01, 144.40, 143.82, 143.63, 52.32, 51.62, 51.53, 30.01, 25.80. ^1^H-NMR (500 MHz, DMSO-*d*_6_): δ 9.21–9.15 (m, 2H), 8.90 (d, *J* = 2.5 Hz, 1H), 8.79–8.74 (m, 1H), 4.62–4.53 (m, 1H), 3.65 (s, 3H), 3.56 (s, 3H), 2.41 (t, *J* = 7.4 Hz, 2H), 2.24–2.15 (m, 1H), 2.14–2.02 (m, 1H). IR (ATR-Ge, cm^−1^): 3377 CONH N-H stretching, 1677 CONH C=O stretching, 1520 CONH C-N stretching, 1737 COOR C=O stretching, 2954 C-H stretching. [α]_D_ = 16.0° (methanol). MS (EI): M^+·^ = 281.

*PC-l-Glu-diEt (diethyl (pyrazine-2-carbonyl)-l-glutamate)*

Oily yellow liquid. Yield: 72%. ^13^C-NMR (126 MHz, DMSO-*d*_6_): δ 172.38, 171.25, 163.41, 147.99, 144.43, 143.79, 143.62, 61.00, 60.08, 51.74, 30.24, 25.82, 14.20. ^1^H-NMR (500 MHz, DMSO-*d*_6_): δ 9.18 (d, *J* = 1.4 Hz, 1H), 9.13 (d, *J* = 8.0 Hz, 1H), 8.90 (d, *J* = 2.5 Hz, 1H), 8.79–8.74 (m, 1H), 4.59–4.51 (m, 1H), 4.17–4.07 (m, 2H), 4.02 (q, *J* = 7.1 Hz, 2H), 2.43–2.36 (m, 2H), 2.23–2.04 (m, 2H), 1.18 (t, *J* = 7.1 Hz, 3H), 1.14 (t, *J* = 7.1 Hz, 3H). IR (ATR-Ge, cm^−1^): 3359 CONH N-H stretching, 1677 CONH C=O stretching, 1520 CONH C-N stretching, 1732 COOR C=O stretching, 2982 C-H stretching. [α]_D_ = −12.0° (methanol). MS (EI): M^+·^ = 309.

*PC-Gly ((pyrazine-2-carbonyl)glycine)*

CAS 57229-37-5. White solid. Yield: 10%. m.p. not measured due to insufficient quantity of the compound, structure confirmed by MS and NMR, (lit. from dimethylformamide, methanol 233–235°C [63], lit. from water, 229 °C [64]). ^13^C-NMR (126 MHz, DMSO-*d*_6_): δ 171.39, 162.70, 147.88, 144.59, 143.69, 143.55, 42.33. ^1^H-NMR (500 MHz, DMSO-*d*_6_): δ 9.18 (s, 1H), 8.93–8.80 (m, 2H), 8.74 (s, 1H), 3.87 (d, *J* = 5.0 Hz, 2H). IR (ATR-Ge, cm^−1^): 3349 CONH N-H and COOH O-H stretching broad band, 1652 CONH C=O stretching, 1717 COOH C=O stretching, 2932 C-H stretching. MS (ESI-): [M−H]^−^ = 180.

*PC-Gly-Et (ethyl (pyrazine-2-carbonyl)glycinate)*

CAS 544702-05-8. White solid. Yield: 22%. m.p. = 92–95 °C (lit. 89–93 [65]). ^13^C-NMR (126 MHz, DMSO-*d*_6_): δ 169.60, 163.54, 148.07, 144.36, 143.74, 143.70, 60.81, 41.23, 14.27. ^1^H-NMR (500 MHz, DMSO-*d*_6_): δ 9.23 (t, *J* = 6.1 Hz, 1H), 9.19 (d, *J* = 1.5 Hz, 1H), 8.90 (d, *J* = 2.5 Hz, 1H), 8.77–8.75 (m, 1H), 4.12 (q, *J* = 7.1 Hz, 2H), 4.05 (d, *J* = 6.1 Hz, 2H), 1.20 (t, *J* = 7.1 Hz, 3H). IR (ATR-Ge, cm^−1^): 3371 CONH N-H stretching, 1666 CONH C=O stretching, 1530 CONH C-N stretching, 1741 COOR C=O stretching, 2986 C-H stretching. MS (EI): M^+·^ = 209.

*PC-d-Ile-Me (methyl (pyrazine-2-carbonyl)-d-isoleucinate)*

Oily yellow liquid. Yield: 38%. ^13^C-NMR (126 MHz, DMSO-*d*_6_): δ 171.82, 163.11, 148.15, 144.13, 143.72, 143.66, 55.58, 52.29, 36.71, 25.83, 14.99, 11.55. ^1^H-NMR (500 MHz, DMSO-*d*_6_): δ 9.19 (d, *J* = 1.5 Hz, 1H), 8.91 (d, *J* = 2.4 Hz, 1H), 8.81–8.74 (m, 1H), 8.56 (d, *J* = 8.8 Hz, 1H), 4.64 (dd, *J* = 8.8, *J* = 5.1 Hz, 1H), 3.69 (s, 3H), 2.13–1.99 (m, 1H), 1.46–1.30 (m, 1H), 1.23–1.09 (m, 1H), 0.93–0.85 (m, 6H). IR (ATR-Ge, cm^−1^): 3390 CONH N-H stretching, 1682 CONH C=O stretching, 1518 CONH C-N stretching, 1741 COOR C=O stretching, 2960 C-H stretching. MS (EI): M^+·^ = 251.

*PC-l-Ile-Me (methyl (pyrazine-2-carbonyl)-l-isoleucinate)*

Oily yellow liquid. Yield: 78%. ^13^C-NMR (126 MHz, DMSO-*d*_6_): δ 171.67, 163.06, 148.12, 144.23, 143.75, 143.67, 56.74, 52.13, 36.38, 25.17, 15.58, 11.18. ^1^H-NMR (500 MHz, DMSO-*d*_6_): δ 9.18 (d, *J* = 1.4 Hz, 1H), 8.91 (d, *J* = 2.5 Hz, 1H), 8.79–8.74 (m, 1H), 8.71 (d, *J* = 8.3 Hz, 1H), 4.48 (dd, *J* = 8.3, *J* = 6.5 Hz, 1H), 3.68 (s, 3H), 2.07–1.98 (m, 1H), 1.53–1.42 (m, 1H), 1.27–1.17 (m, 1H), 0.93–0.83 (m, 6H). IR (ATR-Ge, cm^−1^): 3395 CONH N-H stretching, 1681 CONH C=O stretching, 1519 CONH C-N stretching, 1741 COOR C=O stretching, 2965 C-H stretching. MS (EI): M^+·^ = 251.

*PC-dl-Leu-Et ethyl (pyrazine-2-carbonyl)leucinate)*

CAS 2319608-35-8. Oily yellow liquid. Yield: 83%. ^13^C-NMR (126 MHz, DMSO-*d*_6_): δ 172.05, 163.28, 147.95, 144.49, 143.80, 143.61, 60.85, 50.74, 24.57, 22.99, 21.34, 14.21. ^1^H-NMR (500 MHz, DMSO-*d*_6_): δ 9.18 (d, *J* = 1.3 Hz, 1H), 9.04 (d, *J* = 8.2 Hz, 1H), 8.89 (d, *J* = 2.5 Hz, 1H), 8.79–8.73 (m, 1H), 4.61–4.51 (m, 1H), 4.18–4.05 (m, 2H), 1.95–1.82 (m, 1H), 1.69–1.56 (m, 2H), 1.18 (t, *J* = 7.1 Hz, 3H), 0.89 (d, *J* = 6.1 Hz, 3H), 0.87 (d, *J* = 6.1 Hz, 3H). IR (ATR-Ge, cm^−1^): 3386 CONH N-H stretching, 1675 CONH C=O stretching, 1520 CONH C-N stretching, 1739 COOR C=O stretching, 2959 C-H stretching. MS (EI): M^+·^ = 265.

*PC-d-Leu-Me (methyl (pyrazine-2-carbonyl)-d-leucinate)*

Oily yellow liquid. Yield: 85%. ^13^C-NMR (126 MHz, DMSO-*d*_6_): δ 172.55, 163.30, 147.97, 144.47, 143.83, 143.62, 52.21, 50.62, 24.55, 23.01, 21.31. ^1^H-NMR(500 MHz, DMSO-*d*_6_): δ 9.18 (d, *J* = 1.5 Hz, 1H), 9.09 (d, *J* = 8.3 Hz, 1H), 8.89 (d, *J* = 2.7 Hz, 1H), 8.78–8.73 (m, 1H), 4.63–4.55 (m, 1H), 3.65 (s, 3H), 1.95–1.84 (m, 1H), 1.68–1.56 (m, 2H), 0.89 (d, *J* = 6.0 Hz, 3H), 0.87 (d, *J* = 6.0 Hz, 3H). IR (ATR-Ge, cm^−1^): 3325CONH N-H stretching, 1673 CONH C=O stretching, 1519 CONH C-N stretching, 1743 COOR C=O stretching, 2956 C-H stretching. MS (EI): M^+·^ = 251.

*PC-l-Leu-Me (methyl (pyrazine-2-carbonyl)-l-leucinate)*

CAS 1190303-01-5. Oily yellow liquid. Yield: 66%. ^13^C-NMR (126 MHz, DMSO-*d*_6_): δ 172.54, 163.30, 147.96, 144.47, 143.83, 143.62, 52.21, 50.62, 24.55, 23.01, 21.31. ^1^H-NMR (500 MHz, DMSO-*d*_6_): δ 9.18 (d, *J* = 1.5 Hz, 1H), 9.09 (d, *J* = 8.3 Hz, 1H), 8.89 (d, *J* = 2.5 Hz, 1H), 8.78–8.74 (m, 1H), 4.66–4.53 (m, 1H), 3.65 (s, 3H), 1.95–1.84 (m, 1H), 1.68–1.56 (m, 2H), 0.89 (d, *J* = 6.0 Hz, 3H), 0.87 (d, *J* = 6.0 Hz, 3H). IR (ATR-Ge, cm^−1^): 3325 CONH N-H stretching, 1674 CONH C=O stretching, 1520 CONH C-N stretching, 1742 COOR C=O stretching, 2955 C-H stretching. MS (EI): M^+·^ = 251.

*PC-MeAcr (methyl 2-(pyrazine-2-carboxamido)acrylate)*

CAS 1027723-22-3. White solid. Yield: 11%. m.p. = 102–104 °C (lit. 95.5 °C [32]). ^13^C-NMR (126 MHz, DMSO-*d*_6_): δ 163.80, 161.52, 148.64, 143.79, 143.65, 143.48, 131.57, 109.67, 53.31. ^1^H-NMR (500 MHz, DMSO-*d*_6_): δ 10.10 (bs, 1H), 9.27 (d, *J* = 1.4 Hz, 1H), 8.96 (d, *J* = 2.5 Hz, 1H), 8.82–8.77 (m, 1H), 6.56 (s, 1H), 5.91 (s, 1H), 3.83 (s, 3H). IR (ATR-Ge, cm^−1^): 3349 CONH N-H stretching, 1689 CONH C=O stretching, 1519 CONH C-N stretching, 1712 COOR C=O stretching, 3158, 2957 C-H stretching. MS (ESI-): [M−H]^−^ = 206.

*PC-l-Met-Me (methyl (pyrazine-2-carbonyl)-l-methioninate)*

CAS 1454692-96-6. Oily yellow liquid. Yield: 66%. ^13^C-NMR (126 MHz, DMSO-*d*_6_): δ 171.95, 163.48, 147.97, 144.47, 143.84, 143.61, 52.32, 51.34, 30.10, 29.97, 14.69. ^1^H-NMR (500 MHz, DMSO-*d*_6_): δ 9.21 (d, *J* = 8.1 Hz, 1H), 9.19–9.17 (m, 1H), 8.90 (d, *J* = 2.6 Hz, 1H), 8.78–8.75 (m, 1H), 4.74–4.66 (m, 1H), 3.66 (s, 3H), 2.60–2.42 (m, 2H), 2.23–2.06 (m, 2H), 2.04 (s, 3H). IR (ATR-Ge, cm^−1^): 3376 CONH N-H stretching, 1675 CONH C=O stretching, 1519 CONH C-N stretching, 1741 COOR C=O stretching, 2952 C-H stretching. [α]_D_ = −22.7° (methanol), +33.0° (chloroform) (lit. chloroform 27.8° [65]). MS (EI): M^+·^ = 269.

*PC-d/l-Pgl-Me (methyl 2-phenyl-2-(pyrazine-2-carboxamido)acetate)-enantiomeric mixture*

White solid. m.p. = 92–97 °C (lit. racemic mixture from chloroform and light petroleum 101 °C [43], pure l-enantiomer 53 °C). Yield: 55%. ^13^C-NMR (126 MHz, DMSO-*d*_6_): δ 170.66, 162.68, 148.20, 144.17, 143.75, 143.73, 136.62, 128.85, 128.48, 128.04, 56.35, 52.82. ^1^H-NMR (500 MHz, DMSO-*d*_6_): δ 9.26 (d, *J* = 7.5 Hz, 1H), 9.18 (d, *J* = 1.5 Hz, 1H), 8.91 (d, *J* = 2.5 Hz, 1H), 8.79–8.75 (m, 1H), 7.51–7.45 (m, 2H), 7.43–7.29 (m, 3H), 5.73 (d, *J* = 7.5 Hz, 1H), 3.68 (s, 3H). IR (ATR-Ge, cm^−1^): 3376 CONH N-H stretching, 1665 CONH C=O stretching, 1516 CONH C-N stretching, 1747 COOR C=O stretching, 3090, 2953 C-H stretching. [α]_D_ = –47.3° (chloroform). MS (EI): M^+·^ = 271.

*PC-dl-Phe-Et (ethyl (pyrazine-2-carbonyl)phenylalaninate)*

Brownish solid. Yield: 94%. m.p. = 58–61 °C. ^13^C-NMR (126 MHz, DMSO-*d*_6_): δ 171.08, 163.01, 148.05, 144.21, 143.68, 143.65, 137.37, 129.26, 128.43, 126.72, 61.02, 53.81, 36.33, 14.15. ^1^H-NMR (500 MHz, DMSO-*d*_6_): δ 9.13 (d, *J* = 1.5 Hz, 1H), 9.02 (d, *J* = 8.1 Hz, 1H), 8.88 (d, *J* = 2.5 Hz, 1H), 8.75–8.73 (m, 1H), 7.29–7.21 (m, 4H), 7.20–7.15 (m, 1H), 4.81–4.74 (m, 1H), 4.11 (q, *J* = 7.1 Hz, 2H), 3.27–3.16 (m, 2H), 1.15 (t, *J* = 7.1 Hz, 3H). IR (ATR-Ge, cm^−1^): 3334 CONH N-H stretching, 1660 CONH C=O stretching, 1523 CONH C-N stretching, 1741 COOR C=O stretching, 3064, 2977 C-H stretching. MS (EI): M^+·^ = 299.

*PC-d-Ser ((pyrazine-2-carbonyl)-d-serine)*

CAS 193957-37-8. White solid. Yield: 20%. m.p. = 185–189 °C (carbonized). ^13^C-NMR (126 MHz, DMSO-*d*_6_): δ 171.69, 162.54, 148.11, 144.21, 143.66, 143.56, 61.29, 54.93. ^1^H-NMR (500 MHz, DMSO-*d*_6_): δ 9.23–9.19 (m, 1H), 8.91 (d, *J* = 2.5 Hz, 1H), 8.80–8.75 (m, 1H), 8.68 (d, *J* = 7.9 Hz, 1H), 4.52–4.45 (m, 1H), 3.89 (dd, *J* = 11.1, *J* = 4.0 Hz, 1H), 3.78 (dd, *J* = 11.1, *J* = 3.4 Hz, 1H). IR (ATR-Ge, cm^−1^): 3365 CONH N-H stretching, 1651 CONH C=O stretching, 1537 CONH C-N stretching, 1708 COOH C=O stretching, 3335 CH_2_OH O-H stretching, 2935 C-H stretching. [α]_D_ not measured due to insolubility. MS (ESI-): [M−H]^−^ = 210.

*PC-d-Ser-Me (methyl (pyrazine-2-carbonyl)-d-serinate)*

White solid. Yield: 45%. m.p. = 54–56 °C (Kofler). ^13^C-NMR (126 MHz, DMSO-*d*_6_): δ 170.95, 163.15, 148.52, 144.33, 143.98, 143.95, 61.49, 55.19, 52.64. ^1^H-NMR (500 MHz, DMSO-*d*_6_): δ 9.21 (d, *J* = 1.5 Hz, 1H), 8.93 (d, *J* = 2.5 Hz, 1H), 8.81 (d, *J* = 8.1 Hz, 1H), 8.79 (dd, *J* = 2.5, *J* = 1.5 Hz, 1H), 5.27 (bs, 1H), 4.70–4.56 (m, 1H), 3.93–3.85 (m, 1H), 3.83–3.75 (m, 1H), 3.67 (s, 3H). IR (ATR-Ge, cm^−1^): 1656 CONH C=O stretching, 1542 CONH C-N stretching, 1737 COOR C=O stretching, 3343 CH_2_OH O-H stretching broad band, 2956 C-H stretching. MS (EI): M^+·^ = 225.

*PC-l-Ser-Me (methyl (pyrazine-2-carbonyl)-l-serinate)*

CAS 139934-07-9. White solid. Yield: 59%. m.p. = 56–58 °C (Kofler). ^13^C-NMR (126 MHz, DMSO-*d*_6_): δ 170.66, 162.85, 148.23, 144.04, 143.68, 143.66, 61.20, 54.91, 52.35. ^1^H-NMR (500 MHz, DMSO-*d*_6_): δ 9.21 (d, *J* = 1.5 Hz, 1H), 8.93 (d, *J* = 2.7 Hz, 1H), 8.81 (d, *J* = 8.1 Hz, 1H), 8.79–8.77 (m, 1H), 5.27 (t, *J* = 5.9 Hz, 1H), 4.66–4.59 (m, 1H), 3.93–3.86 (m, 1H), 3.84–3.76 (m, 1H), 3.68 (s, 3H). IR (ATR-Ge, cm^−1^): 1657 CONH C=O stretching, 1543 CONH C-N stretching, 1737 COOR C=O stretching, 3340 CH_2_OH O-H stretching broad band, 2956 C-H stretching. MS (EI): M^+·^ = 225.

*PC-l-OBn-Ser (O-benzyl-N-(pyrazine-2-carbonyl)-l-serine)*

White solid. Yield: 50%. m.p. = 139–142 °C. ^13^C-NMR (126 MHz, DMSO-*d*_6_): δ 171.20, 162.58, 148.21, 144.00, 143.71, 143.60, 138.12, 128.41, 127.70, 127.66, 72.30, 69.20, 52.61. ^1^H-NMR (500 MHz, DMSO-*d*_6_): δ 13.14 (s, 1H), 9.21 (s, 1H), 8.93 (s, 1H), 8.78 (s, 1H), 8.73 (d, *J* = 8.2 Hz, 1H), 7.37–7.22 (m, 5H), 4.80–4.70 (m, 1H), 4.53 (s, 2H), 4.05–3.91 (m, 1H), 3.88–3.79 (m, 1H). IR (ATR-Ge, cm^−1^): 3400 CONH N-H stretching, 1650 CONH C=O stretching, 1542 CONH C-N stretching, 1743 COOH C=O stretching, 2943 COOH O-H stretching broad band, 2935 C-H stretching. [α]_D_ = 45.3° (methanol). MS (ESI-): [M−H]^−^ = 300.

*PC-l-OBn-Ser-Me (methyl O-benzyl-N-(pyrazine-2-carbonyl)-l-serinate)*

Oily yellowish liquid. Yield: 95%. ^13^C-NMR (126 MHz, DMSO-*d*_6_): δ 170.53, 163.14, 148.56, 144.23, 144.02, 143.97, 138.32, 128.74, 128.04, 127.99, 72.51, 69.12, 52.83, 52.79. ^1^H-NMR (500 MHz, DMSO-*d*_6_): δ 9.20 (d, *J* = 1.5 Hz, 1H), 8.92 (d, *J* = 2.5 Hz, 1H), 8.88 (d, *J* = 8.2 Hz, 1H), 8.81–8.75 (m, 1H), 7.35–7.28 (m, 2H), 7.31–7.23 (m, 3H), 4.88–4.81 (m, 1H), 4.55 (d, *J* = 12.2 Hz, 1H), 4.50 (d, *J* = 12.2 Hz, 1H), 3.93 (dd, *J* = 9.9, 5.5 Hz, 1H), 3.84 (dd, *J* = 10.0, 4.0 Hz, 1H), 3.67 (s, 3H). IR (ATR-Ge, cm^−1^): 3398 CONH N-H stretching, 1679 CONH C=O stretching, 1519CONH C-N stretching, 1746 COOR C=O stretching, 2921 C-H stretching. [α]_D_ = 10.7° (methanol). MS (EI): M^+·^ = 315.

*PC-d-OtBu-Ser-Me (methyl O-(tert-butyl)-N-(pyrazine-2-carbonyl)-d-serinate)*

Oily yellow liquid. Yield: 100%. ^13^C-NMR (126 MHz, DMSO-*d*_6_): δ 170.43, 162.62, 148.32, 143.83, 143.78, 143.63, 73.33, 61.54, 53.00, 52.40, 27.28. ^1^H-NMR (500 MHz, DMSO-*d*_6_): δ 9.21 (d, *J* = 1.5 Hz, 1H), 8.92 (d, *J* = 2.5 Hz, 1H), 8.81–8.76 (m, 1H), 8.62 (d, *J* = 8.3 Hz, 1H), 4.76–4.69 (m, 1H), 3.83 (dd, *J* = 9.6, 4.4 Hz, 1H), 3.70 (dd, *J* = 9.6, 3.9 Hz, 1H), 3.68 (s, 3H), 1.11 (s, 9H). IR (ATR-Ge, cm^−1^): 3404 CONH N-H stretching, 1681 CONH C=O stretching, 1519 CONH C-N stretching, 1748 COOR C=O stretching, 2973 C-H stretching. MS (EI): M^+·^ = 281.

*PC-l-OtBu-Ser-Me (methyl O-(tert-butyl)-N-(pyrazine-2-carbonyl)-l-serinate)*

Oily yellow liquid. Yield: 61%. ^13^C-NMR (126 MHz, DMSO-*d*_6_) 170.43, 162.62, 148.32, 143.84, 143.78, 143.64, 73.33, 61.55, 53.01, 52.39, 27.28. ^1^H-NMR (500 MHz, DMSO-*d*_6_): δ 9.21 (d, *J* = 1.5 Hz, 1H), 8.92 (d, *J* = 2.5 Hz, 1H), 8.80–8.76 (m, 1H), 8.62 (d, *J* = 8.3 Hz, 1H), 4.76–4.69 (m, 1H), 3.83 (dd, *J* = 9.6, 4.3 Hz, 1H), 3.70 (dd, *J* = 9.6, 3.9 Hz, 1H), 3.68 (s, 3H), 1.10 (s, 9H). IR (ATR-Ge, cm^−1^): 3404 CONH N-H stretching, 1681 CONH C=O stretching, 1518 CONH C-N stretching, 1747 COOR C=O stretching, 2973 C-H stretching. MS (EI): M^+·^ = 281.

*PC-d-Thr-Me (methyl (pyrazine-2-carbonyl)-d-threoninate)*

Oily yellow liquid. Yield: 95%. ^13^C-NMR (126 MHz, DMSO-*d*_6_): δ 170.84, 163.12, 148.37, 143.80, 143.75, 143.66, 66.46, 57.99, 52.37, 20.57. ^1^H-NMR (500 MHz, DMSO-*d*_6_): δ 9.22 (d, *J* = 1.5 Hz, 1H), 8.94 (d, *J* = 2.5 Hz, 1H), 8.82–8.78 (m, 1H), 8.54 (d, *J* = 8.9 Hz, 1H), 4.53 (dd, *J* = 8.9, *J* = 2.9 Hz, 1H), 4.27 (qd, *J* = 6.4, *J* = 2.9 Hz, 1H), 3.79 (bs, 1H), 3.68 (s, 3H), 1.12 (d, *J* = 6.4 Hz, 3H). IR (ATR-Ge, cm^−1^): 1671 CONH C=O stretching, 1525 CONH C-N stretching, 1741 COOR C=O stretching, 3387 CH_2_OH O-H stretching broad band, 2955 C-H stretching. MS (EI): M^+·^ = 239 not visible in GC-MS spectrum, confirmed by MS (ESI-) [M−H]^−^ = 240.

*PC-l-Thr-Me (methyl (pyrazine-2-carbonyl)-l-threoninate)*

Brownish solid. Yield: 90%. m.p. = 69–72 °C. ^13^C-NMR (126 MHz, DMSO-*d*_6_): δ 170.83, 163.10, 148.36, 143.79, 143.74, 143.65, 66.44, 57.98, 52.36, 20.56. ^1^H-NMR (500 MHz, DMSO-*d*_6_): δ 9.22 (d, *J* = 1.5 Hz, 1H), 8.94 (d, *J* = 2.5 Hz, 1H), 8.82–8.78 (m, 1H), 8.55 (d, *J* = 8.9 Hz, 1H), 5.40 (bs, 1H), 4.54 (dd, *J* = 8.9, *J* = 2.8 Hz, 1H), 4.27 (qd, *J* = 6.4, *J* = 2.9 Hz, 1H), 3.68 (s, 3H), 1.12 (d, *J* = 6.4 Hz, 3H). IR (ATR-Ge, cm^−1^): 1664 CONH C=O stretching, 1527 CONH C-N stretching, 1744 COOR C=O stretching, 3380 CH_2_OH O-H stretching broad band, 2981 C-H stretching. MS (EI): M^+·^ = 239 not visible, same GC-MS spectrum as PC-d-Thr-Me.

*PC-d-OtBu-Thr-Me (methyl O-(tert-butyl)-N-(pyrazine-2-carbonyl)-d-threoninate)*

White solid. Yield: 89%. m.p. = 81–84 °C. ^13^C-NMR (126 MHz, DMSO-d_6_): δ 170.61, 162.90, 148.48, 143.84, 143.61, 143.52, 74.02, 67.22, 57.70, 52.37, 28.22, 21.01. ^1^H-NMR (500 MHz, DMSO-*d*_6_): δ 9.23 (d, *J* = 1.5 Hz, 1H), 8.95 (d, *J* = 2.5 Hz, 1H), 8.81–8.80 (m, 1H), 8.29 (d, *J* = 9.2 Hz, 1H), 4.58 (dd, *J* = 9.2, *J* = 2.2 Hz, 1H), 4.32 (qd, *J* = 6.2, *J* = 2.2 Hz, 1H), 3.68 (s, 3H), 1.14 (d, *J* = 6.2 Hz, 3H), 1.11 (s, 9H). IR (ATR-Ge, cm^−1^): 3411 CONH N-H stretching, 1685 CONH C=O stretching, 1519 CONH C-N stretching, 1743 COOR C=O stretching, 2977 C-H stretching. MS (EI): M^+·^ = 295 not visible in GC-MS spectrum, confirmed by MS (ESI-) [M−H]^−^ = 294.

*PC-l-OtBu-Thr-Me (methyl O-(tert-butyl)-N-(pyrazine-2-carbonyl)-l-threoninate)*

White solid. Yield: 99%. m.p. = 83–86 °C. ^13^C-NMR (126 MHz, DMSO-*d*_6_): δ 170.60, 162.90, 148.47, 143.83, 143.60, 143.51, 74.01, 67.22, 57.69, 52.36, 28.21, 21.00. ^1^H-NMR (500 MHz, DMSO-*d*_6_): δ 9.23 (d, *J* = 1.5 Hz, 1H), 8.95 (d, *J* = 2.4 Hz, 1H), 8.83–8.78 (m, 1H), 8.29 (d, *J* = 9.2 Hz, 1H), 4.58 (dd, *J* = 9.2, *J* = 2.2 Hz, 1H), 4.32 (qd, *J* = 6.2, *J* = 2.2 Hz, 1H), 3.68 (s, 3H), 1.14 (d, *J* = 6.2 Hz, 3H), 1.11 (s, 9H). IR (ATR-Ge, cm^−1^): 3410 CONH N-H stretching, 1685 CONH C=O stretching, 1518 CONH C-N stretching, 1743 COOR C=O stretching, 2977 C-H stretching. MS (EI): M^+·^ = 295 not visible, same GC-MS spectrum as PC-d-*Ot*Bu-Thr-Me.

*PC-d-Trp-Et (ethyl (pyrazine-2-carbonyl)-d-tryptophanate)*

White solid. Yield: 99%. m.p. = 90–93 °C. ^13^C-NMR (126 MHz, DMSO-*d*_6_): δ 171.42, 162.90, 148.05, 144.20, 143.67, 143.60, 136.30, 127.31, 123.97, 121.20, 118.57, 118.25, 111.62, 109.41, 61.03, 53.36, 26.84, 14.14. ^1^H-NMR (500 MHz, DMSO-*d*_6_): δ 10.85 (s, 1H), 9.15 (d, *J* = 1.4 Hz, 1H), 8.91–8.85 (m, 2H), 8.73–8.69 (m, 1H), 7.50 (d, *J* = 8.1 Hz, 1H), 7.32 (d, *J* = 8.1 Hz, 1H), 7.17 (d, *J* = 2.4 Hz, 1H), 7.08–7.01 (m, 1H), 6.98–6.90 (m, 1H), 4.85–4.77 (m, 1H), 4.10 (q, *J* = 7.1 Hz, 2H), 3.41–3.29 (m, 2H), 1.14 (t, *J* = 7.1 Hz, 3H). IR (ATR-Ge, cm^−1^): 3366 CONH N-H stretching, 1673 CONH C=O stretching, 1520 CONH C-N stretching, 1732 COOR C=O stretching, 2980 C-H stretching. MS (EI): M^+·^ = 338.

*PC-l-Trp-Et (ethyl (pyrazine-2-carbonyl)-l-tryptophanate)*

Oily yellow liquid. Yield: 96%. ^13^C-NMR (126 MHz, DMSO-*d*_6_): δ 171.42, 162.90, 148.05, 144.20, 143.67, 143.60, 136.30, 127.31, 123.97, 121.19, 118.57, 118.25, 111.62, 109.40, 61.03, 53.36, 26.84, 14.13. ^1^H-NMR (500 MHz, DMSO-*d*_6_): δ 10.86 (s, 1H), 9.16 (d, *J* = 1.5 Hz, 1H), 8.89 (d, *J* = 7.7 Hz, 1H), 8.88 (d, *J* = 2.5 Hz, 1H), 8.73–8.69 (m, 1H), 7.50 (d, *J* = 8.0 Hz, 1H), 7.32 (d, *J* = 8.0 Hz, 1H), 7.18 (d, *J* = 2.3 Hz, 1H), 7.10–6.97 (m, 1H), 6.97–6.91 (m, 1H), 4.85–4.78 (m, 1H), 4.10 (q, *J* = 7.1 Hz, 2H), 3.40–3.30 (m, 2H), 1.14 (t, *J* = 7.1 Hz, 3H). IR (ATR-Ge, cm^−1^): 3368 CONH N-H stretching, 1672 CONH C=O stretching, 1520 CONH C-N stretching, 1732 COOR C=O stretching, 2981 C-H stretching. MS (EI): M^+·^ = 338.

*PC-d-Tyr-Me (methyl (pyrazine-2-carbonyl)-d-tyrosinate)*

Oily yellow liquid. Yield: 99%. ^13^C-NMR (126 MHz, DMSO-*d*_6_): δ 171.70, 162.95, 156.18, 148.08, 144.19, 143.70, 143.68, 130.20, 127.26, 115.30, 54.02, 52.27, 35.58. ^1^H-NMR (500 MHz, DMSO-*d*_6_): δ 9.21 (s, 1H), 9.13 (d, *J* = 1.5 Hz, 1H), 8.94 (d, *J* = 8.1 Hz, 1H), 8.88 (d, *J* = 2.5 Hz, 1H), 8.75–8.73 (m, 1H), 7.03–6.98 (m, AA’, BB’, 2H), 6.66–6.60 (m, AA’, BB’, 2H), 4.81–4.66 (m, 1H), 3.65 (s, 3H), 3.09 (d, *J* = 7.0 Hz, 2H). IR (ATR-Ge, cm^−1^): 3351 CONH N-H stretching, 1669 CONH C=O stretching, 1515 CONH C-N stretching, 1736 COOR C=O stretching, 2953 C-H stretching. [α]_D_ = 12.0° (methanol). MS (ESI-): [M−H]^−^ = 300.

*PC-l-Tyr-Et (ethyl (pyrazine-2-carbonyl)-l-tyrosinate)*

White solid. Yield: 65%. m.p. = 119–123 °C. ^13^C-NMR (126 MHz, DMSO-*d*_6_): δ 171.21, 162.92, 156.20, 148.07, 144.21, 143.68, 143.67, 130.23, 127.19, 115.27, 60.99, 54.09, 35.66, 14.19. ^1^H-NMR (500 MHz, DMSO-*d*_6_): δ 9.21 (s, 1H), 9.14 (d, *J* = 1.3 Hz, 1H), 8.92–8.86 (m, 2H), 8.76–8.73 (m, 1H), 7.03–6.98 (m, AA’, BB’, 2H), 6.66–6.60 (m, AA’, BB’, 2H), 4.73–4.65 (m, 1H), 4.11 (q, *J* = 7.1 Hz, 2H), 3.09 (dd, *J* = 7.0, 2.7 Hz, 2H), 1.16 (t, *J* = 7.1 Hz, 3H). IR (ATR-Ge, cm^−1^): 3367 CONH N-H stretching, 1663 CONH C=O stretching, 1519 CONH C-N stretching, 1720 COOR C=O stretching, 3440 Ar-OH O-H broad band, 2986 C-H stretching. [α]_D_ = -5.0° (methanol). MS (ESI-): [M−H]^−^ = 314.

*PC-dl-Val-Et (ethyl (pyrazine-2-carbonyl)valinate)*

Oily yellow liquid. Yield: 31%. ^13^C-NMR (126 MHz, DMSO-*d*_6_): δ 171.12, 163.11, 148.12, 144.23, 143.73, 143.67, 60.94, 57.77, 30.21, 19.10, 18.49, 14.25. ^1^H-NMR (500 MHz, DMSO-*d*_6_): δ 9.18 (d, *J* = 1.3 Hz, 1H), 8.91 (d, *J* = 2.6 Hz, 1H), 8.80–8.75 (m, 1H), 8.64 (d, *J* = 8.4 Hz, 1H), 4.41 (dd, *J* = 8.4, *J* = 6.3 Hz, 1H), 4.21–4.10 (m, 2H), 2.32–2.19 (m, 1H), 1.20 (t, *J* = 7.1 Hz, 3H), 0.94 (d, *J* = 6.8 Hz, 3H), 0.93 (d, *J* = 6.8 Hz, 3H). IR (ATR-Ge, cm^−1^): 3395 CONH N-H stretching, 1682 CONH C=O stretching, 1519 CONH C-N stretching, 1737 COOR C=O stretching, 2966 C-H stretching. MS (EI): M^+·^ = 251.

*PC-d-Val-Me (methyl (pyrazine-2-carbonyl)-d-valinate)*

Oily yellow liquid. Yield: 34%. ^13^C-NMR (126 MHz, DMSO-*d*_6_): δ 171.64, 163.15, 148.11, 144.24, 143.75, 143.67, 57.79, 52.16, 30.13, 19.15, 18.53. ^1^H-NMR (500 MHz, DMSO-*d*_6_): δ 9.18 (d, *J* = 1.5 Hz, 1H), 8.91 (d, *J* = 2.5 Hz, 1H), 8.78–8.76 (m, 1H), 8.69 (d, *J* = 8.4 Hz, 1H), 4.43 (dd, *J* = 8.4, 6.4 Hz, 1H), 3.68 (s, 3H), 2.36–2.16 (m, 1H), 0.93 (dd, *J* = 6.8 Hz, 3H), 0.92 (d, *J* = 6.8 Hz, 3H). IR (ATR-Ge, cm^−1^): 3394 CONH N-H stretching, 1681 CONH C=O stretching, 1519 CONH C-N stretching, 1740 COOR C=O stretching, 2964 C-H stretching. MS (EI): M^+·^ = 237.

*PC-l-Val-Me (methyl (pyrazine-2-carbonyl)-l-valinate)*

CAS 73058-33-0. Oily yellow liquid. Yield: 76%. ^13^C-NMR (126 MHz, DMSO-*d*_6_): δ 171.64, 163.15, 148.11, 144.24, 143.76, 143.66, 57.80, 52.16, 30.14, 19.15, 18.54. ^1^H-NMR(500 MHz, DMSO-*d*_6_): δ 9.18 (d, *J* = 1.5 Hz, 1H), 8.91 (d, *J* = 2.6 Hz, 1H), 8.79–8.75 (m, 1H), 8.69 (d, *J* = 8.5 Hz, 1H), 4.43 (dd, *J* = 8.5, *J* = 6.4 Hz, 1H), 3.68 (s, 3H), 2.32–2.19 (m, *J* = 6.8 Hz, 1H), 0.93 (d, *J* = 6.8 Hz, 3H), 0.92 (d, *J* = 6.8 Hz, 3H). IR (ATR-Ge, cm^−1^): 3395 CONH N-H stretching, 1681 CONH C=O stretching, 1519 CONH C-N stretching, 1741 COOR C=O stretching, 2965 C-H stretching. MS (EI): M^+·^ = 237.

### 3.3. Biological Evaluation

#### 3.3.1. Antimycobacterial Activity Screening Against Mtb H37Ra, *M. smegmatis, M. aurum*

Microdilution assay (Microplate Alamar Blue Assay, MABA) [45]. The antimycobacterial assay was performed with fast-growing *M. smegmatis* DSM 43465 (ATCC 607) and *M. aurum* DSM 43999 (ATCC 23366) from the German Collection of Microorganisms and Cell Cultures (Braunschweig, Germany) and with the avirulent strain of Mtb H37Ra ITM-M006710 (ATCC 9431) from Belgian Co-ordinated Collections of Micro-organisms (Antwerp, Belgium). The technique used for activity determination was microdilution broth panel method using 96-well microtitration plates. The culturing medium was Middlebrook 7H9 broth (Sigma-Aldrich) with a defined pH of 6.6 ± 0.2 enriched with 0.4% of glycerol (Sigma-Aldrich) and 10% of Middlebrook OADC growth supplement (Himedia, Mumbai, India). In the case of screening in pH 6, the pH was achieved by addition of 1M HCl (Penta, Prague, Czech Republic).

The mycobacterial strains were cultured on Middlebrook 7H9 agar and suspensions were prepared in Middlebrook 7H9 broth. The final density was adjusted to value 1.0 according to McFarland scale and diluted in the ratio 1:20 (for fast growing mycobacteria) or 1:10 (for Mtb H37Ra) with broth.

The tested compounds were dissolved in dimethyl sulfoxide (DMSO) (Sigma-Aldrich), then Middlebrook 7H9 broth was added to obtain a concentration of 2000 µg/mL. The standards used for activity determination were isoniazid (INH), rifampicin (RIF), and ciprofloxacin (CPX) (Sigma-Aldrich). The final concentrations were reached by binary dilution and the addition of mycobacterial suspension and were set as 500, 250, 125, 62.5, 31.25, 15.625, 7.81, and 3.91 µg/mL. The final concentration of DMSO in any well did not exceeded 2.5% (*v*/*v*) and did not affect the growth of mycobacteria. Positive (broth, DMSO, bacteria) and negative (broth, DMSO) controls were included.

The plates were sealed with polyester adhesive film and incubated in dark at 37 °C without agitation. The addition of 0.01% solution of resazurin sodium salt followed after 48 h of incubation for *M. smegmatis*, after 72 h of incubation for *M. aurum,* and after 120 h of incubation for Mtb H37Ra, respectively. Stain was prepared by dissolving resazurin sodium salt (Sigma-Aldrich) in deionised water to get 0.02% solution. Then, 10% aqueous solution of Tween 80 (Sigma-Aldrich) was prepared. Both liquids were mixed up making use of the same volumes and filtered through a 0.2 µm syringe membrane filter. The microtitration panels were then incubated for further 2.5 h for the determination of activity against *M. smegmatis*, 4 h for *M. aurum*, and 18 h for Mtb H37Ra, respectively.

Antimycobacterial activity was expressed as the minimum inhibitory concentration (MIC). The MIC (in µg/mL) was determined on the basis of stain colour change (blue colour—active; pink colour—not active). The MIC values for standards are presented in the respective tables. All experiments were conducted in duplicate.

#### 3.3.2. Antibacterial and Antifungal Activity Screening

For details, refer to the Supplementary Material.

#### 3.3.3. Cytotoxicity Screening

The human hepatocellular liver carcinoma cell line HepG2 purchased from Health Protection Agency Culture Collections (ECACC, Salisbury, UK) was cultured in MEM (Minimum Essentials Eagle Medium) (Sigma-Aldrich) supplemented with 10% foetal bovine serum (PAA Laboratories GmbH, Pasching, Austria), 1% l-glutamine solution (Sigma-Aldrich), and non-essential amino acid solution (Sigma-Aldrich) in a humidified atmosphere containing 5% CO_2_ at 37 °C. For subculturing, the cells were harvested after trypsin/EDTA (Sigma-Aldrich) treatment at 37 °C. To evaluate cytotoxicity, the cells treated with the tested substances were used as experimental groups, whereas untreated HepG2 cells served as controls.

The cells were seeded in a density of 10,000 cells per well in a 96-well plate. During the next day, the cells were treated with each of the tested substances dissolved in DMSO. The tested substances were prepared at different incubation concentrations (1–1000 µM) in triplicates according to their solubility. Simultaneously, the controls representing 100% cell viability, 0% cell viability (the cells treated with 10% DMSO), no cell control, and vehiculum controls were also prepared in triplicates. After 24 h of incubation in a humidified atmosphere containing 5% CO_2_ at 37 °C, the reagent from the kit CellTiter 96 AQueous One Solution Cell Proliferation Assay (CellTiter 96; PROMEGA, Fitchburg, WI, USA) was added. After 2 h of incubation at 37 °C, the absorbance of the samples was recorded at 490 nm (TECAN, Infinita M200, Austria). A standard toxicological parameter IC_50_ was calculated by nonlinear regression from a semilogarithmic plot of incubation concentration versus the percentage of absorbance relative to untreated controls using GraphPad Prism 8 software (GraphPad Software, San Diego, CA, USA). The compounds were tested in two separate experiments in cell line passage 3 and 23.

## 4. Conclusions

To conclude, thirty-nine *N*-pyrazinoyl aa derivatives were synthesized in this study. Twenty-four of these compounds (mostly d-enantiomers) had not been previously described in the literature (to date 10th February 2020; racemic mixtures and enantiomeric mixtures were considered unique compounds). The in vitro antimicrobial activity of all compounds was investigated, focused on antimycobacterial, antifungal, and antibacterial activity. No activity was detected against any fungal strains. For antibacterial activities, low activity against *Staphylococcus aureus* was noted for PC-l-*Ot*Bu-Thr-Me (MIC = 31.25 µM). The main biological evaluation was thus focused on antimycobacterial testing. The high activity of some compounds was mainly observed against Mtb H37Ra. Significant activity was noted only in the mildly acidic medium of pH 6, whereas, in the commonly utilized pH 6.6, the compounds were generally inactive. The mixture of enantiomers PC-d/l-Pgl-Me presented the best activity (MIC < 1.95 µg/mL, < 7.3 µM, pH 6), which—in molar concentrations—represents four times higher activity than the first-line antitubercular drug PZA. Esters PC-l-Ala-Et, PC-l-Met-Me, PC-l-*O*Bn-Ser-Me and PC-l-Tyr-Et were up to two times more active than PZA (in molar concentrations at pH 6). The derivatives with the free carboxylic group were generally inactive. Based on the observed structure–activity relationships (SAR), we conclude that the activity is tightly linked to the lipophilicity of the compounds (more lipophilic preferred) as well as to the stereochemistry (l-isomers preferred). For the first time, PZA derivatives containing d-aa were investigated. However, their activity was significantly lower compared to the l-enantiomers.

The difference in antimycobacterial activity between l- and d-isomers might indicate the presence of a specific subcellular target for unhydrolyzed product (pharmacodynamic aspect) or simply the faster hydrolysis of the amidic bond of l-isomers by mycobacterial amidases to release the active POA from its prodrug transport form (pharmacokinetics aspect). The exact active species (parent compounds vs. hydrolysed POA) and its MoA on the molecular level are to be elucidated. However, based on the recent biochemical and crystallographic investigations of PanD [12], the most prominent target of PZA, we conclude that our compounds do not act in their non-hydrolysed form against this target, as the binding cavity at the active site of PanD is too small to accommodate anything bigger than POA or 6-Cl-POA.

The work presented in this article opens the door for further medicinal chemistry optimizations of the most active compounds (PC-l-Ala-Et, PC-dl-Leu-Et, PC-l-Met-Me, PC-d/l-Pgl-Me, PC-l-*O*Bn-Ser-Me) and encourages future research. For the next step, we propose the assessment of derivatives combining aa with antimycobacterially active 5-chloropyrazinamide (5-Cl-PZA) as a simplest PZA derivative with known MoA [66]. Our preliminary results show that the l-Leu methyl ester derivative of 5-Cl-PZA is active against Mtb H37Ra (MIC at pH 6.6 was 62.5 µg/mL, at pH 6 it was 3.91 µg/mL). As 5-Cl-PZA is more lipophilic than PZA, even the methyl ester is probably sufficient to effectively penetrate mycobacterial membrane. We thus concluded that the lipophilicity of the aa part of our compounds indeed plays a role in their activity. For a conclusive description of the structure–activity relationships (SAR) of the proposed compounds, more derivatives need to be synthesized focusing mainly on the differences between the activity of different enantiomers with the same ester. This could bring new interesting knowledge of Mtb enzymatic patterns usable also in other applications.

The design of aa-derived small molecules—notably as hydrolysable prodrugs—could be an interesting therapeutic approach for mycobacterial infections. Mtb as any other bacterium is dependent on nitrogen uptake to synthesize vital biomolecules. However, Mtb has been found to prefer proteinogenic aa as the source of nitrogen over ammonium chloride when cultured in vitro [67,68]. Therefore, if the appropriate mechanisms and enzymes involved in these metabolic pathways were identified, the use of aa derived prodrugs could ‘trick’ Mtb to cleave off the aa and at the same time release the active form of the drug inside the cell. The plausibility of this strategy can be rationalized based on the recent discoveries of several selective Mtb proteases essential either for the survival or virulence of Mtb [69,70], which are proposed as good druggable targets. The ability to uptake all proteinogenic aa has been demonstrated, which could help smaller aa-containing (pro)drugs to pass through the mycobacterial membrane barrier [68]. Penetration through the Mtb membrane barrier could perhaps also be managed by one of the known Mtb ATP-binding cassette (ABC) peptide importers, e.g., dipeptide importer DppABCD or oligopeptide importer OppABCD [71]. Notably, the specificity of the latter has been shown to be very broad [72], which could support our speculations.

## Figures and Tables

**Table 1:** Structures of the synthesized compounds.

Code	Structure
PC-d-Ala	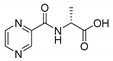
PC-l-Ala	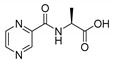
PC-dl-Ala-Me	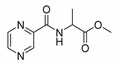
PC-d-Ala-Et	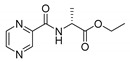
PC-l-Ala-Et	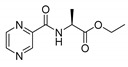
PC-d-Asp-diMe	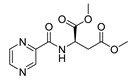
PC-l-Asp-diEt	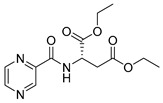
PC-l-*S*Bn-Cys	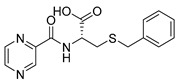
PC-d-Glu-diMe	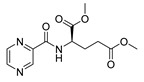
PC-l-Glu-diEt	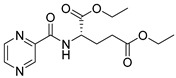
PC-Gly	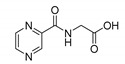
PC-Gly-Et	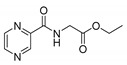
PC-d-Ile-Me	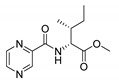
PC-l-Ile-Me	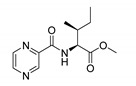
PC-dl-Leu-Et	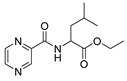
PC-d-Leu-Me	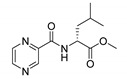
PC-l-Leu-Me	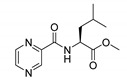
PC-MeAcr	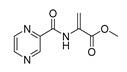
PC-l-Met-Me	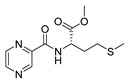
PC-d/l-Pgl-Me	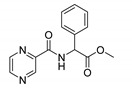
PC-dl-Phe-Et	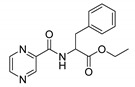
PC-d-Ser	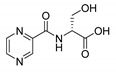
PC-d-Ser-Me	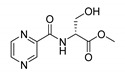
PC-l-Ser-Me	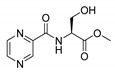
PC-l-*O*Bn-Ser	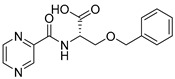
PC-l-*O*Bn-Ser-Me	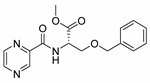
PC-d-*Ot*Bu-Ser-Me	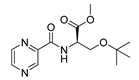
PC-l-*Ot*Bu-Ser-Me	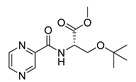
PC-d-Thr-Me	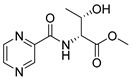
PC-l-Thr-Me	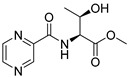
PC-d-*Ot*Bu-Thr-Me	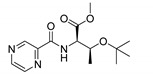
PC-l-*Ot*Bu-Thr-Me	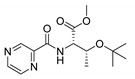
PC-d-Trp-Et	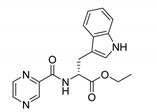
PC-l-Trp-Et	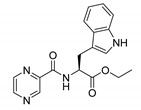
PC-d-Tyr-Me	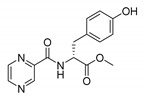
PC-l-Tyr-Et	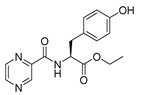
PC-dl-Val-Et	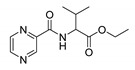
PC-d-Val-Me	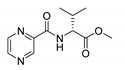
PC-l-Val-Me	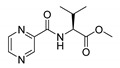

**Table 2:** Antimycobacterial activities of the tested compounds at pH 6.6 presented as the minimum inhibitory concentration (MIC) (µg/mL), the most active compound is in bold. The chemical structures of the derivatives are presented in Table 1.

Code	Mtb H37Ra	*M. smegmatis*	*M. aurum*
PC-d-Ala	≥ 500	≥ 500	≥ 500
PC-l-Ala	≥ 250	≥ 250	≥ 250
PC-dl-Ala-Me	≥ 500	≥ 500	≥ 500
PC-d-Ala-Et	≥ 500	≥ 500	≥ 500
PC-l-Ala-Et	≥ 500	≥ 500	≥ 500
PC-d-Asp-diMe	≥ 125	≥ 125	≥ 125
PC-l-Asp-diEt	≥ 500	≥ 500	≥ 500
PC-l-*S*Bn-Cys	≥ 250	≥ 250	≥ 250
PC-d-Glu-diMe	≥ 500	≥ 500	≥ 500
PC-l-Glu-diEt	≥ 500	≥ 500	≥ 500
PC-Gly	≥ 500	≥ 500	≥ 500
PC-Gly-Et	≥ 500	250	≥ 500
PC-d-Ile-Me	≥ 500	≥ 500	≥ 500
PC-l-Ile-Me	≥ 500	≥ 500	≥ 500
PC-dl-Leu-Et	250	250	250
PC-d-Leu-Me	≥ 500	250	≥ 500
PC-l-Leu-Me	250	250	≥ 500
**PC-MeAcr**	**15.625**	**31.25**	62.5
PC-l-Met-Me	≥ 500	≥ 500	≥ 500
PC-d/l-Pgl-Me	≥ 500	≥ 500	≥ 500
PC-dl-Phe-Et	250	250	≥ 500
PC-d-Ser	≥ 500	≥ 500	≥ 500
PC-d-Ser-Me	≥ 500	≥ 500	≥ 500
PC-l-Ser-Me	≥ 500	≥ 500	≥ 500
PC-l-*O*Bn-Ser	≥ 500	≥ 500	≥ 500
PC-l-*O*Bn-Ser-Me	250	≥ 500	≥ 500
PC-d-*Ot*Bu-Ser-Me	≥ 500	≥ 500	≥ 500
PC-l-*Ot*Bu-Ser-Me	≥ 500	≥ 500	≥ 500
PC-d-Thr-Me	≥ 500	≥ 500	≥ 500
PC-l-Thr-Me	≥ 500	≥ 500	≥ 500
PC-d-*Ot*Bu-Thr-Me	≥ 500	≥ 500	≥ 500
PC-l-*Ot*Bu-Thr-Me	≥ 500	≥ 500	≥ 500
PC-d-Trp-Et	125	250	250
PC-l-Trp-Et	125	250	125
PC-d-Tyr-Me	≥ 500	≥ 500	≥ 500
PC-l-Tyr-Et	500	≥ 500	≥ 500
PC-dl-Val-Et	≥ 500	250	≥ 500
PC-d-Val-Me	≥ 500	≥ 500	≥ 500
PC-l-Val-Me	≥ 500	250	≥ 500
PZA	≥ 500	≥ 500	≥ 500
INH	0.125–0.25	7.81–15.625	1.95–3.91
RIF	0.0039–0.0078	12.5–25	0.39–0.78
CPX	0.125–0.25	0.0625–0.125	0.0078–0.0156

PZA—pyrazinamide, INH—isoniazid, RIF—rifampicin, CPX—ciprofloxacin.

**Table 3:** Antimycobacterial activities of the tested compounds at pH 6 presented as MIC values, most active compounds are in bold. The chemical structures of the derivatives are presented in Table 1.

Code	Mtb H37Ra	Mtb H37Ra	*M. smegmatis*	*M. aurum*	logP **
	µg/mL	µM *	µg/mL	µg/mL	
PC-d-Ala	≥ 125	≥ 640.4	≥ 125	62.5	−1.0155
PC-l-Ala	≥ 250	≥ 1280.9	125	62.5	−1.0155
PC-dl-Ala-Me	≥ 125	≥ 597.5	≥ 125	≥ 125	−0.8725
PC-d-Ala-Et	≥ 125	≥ 560.0	≥ 125	≥ 125	−0.5315
**PC-l-Ala-Et**	**3.91**	**17.5**	≥ 125	≥ 125	−0.5315
PC-d-Asp-diMe	≥ 125	≥ 467.7	≥ 125	≥ 125	−1.3565
PC-l-Asp-diEt	≥ 125	≥ 423.3	≥ 125	≥ 125	−0.6745
PC-l-*S*Bn-Cys	62.5	196.9	≥ 250	125	0.9855
PC-d-Glu-diMe	≥ 500	≥ 1777.7	≥ 500	≥ 500	−0.9145
**PC-l-Glu-diEt**	**31.25**	**101.0**	≥ 500	≥ 500	−0.2325
PC-Gly	≥ 125	≥ 690.0	≥ 125	≥ 125	−1.4775
PC-Gly-Et	≥ 125	≥ 597.5	≥ 125	≥ 125	−0.9935
PC-d-Ile-Me	250	994.9	250	250	0.5415
PC-l-Ile-Me	250	994.9	≥ 500	≥ 500	0.5415
**PC-dl-Leu-Et**	**15.625**	**58.9**	≥ 125	≥ 125	0.8825
PC-d-Leu-Me	≥ 125	≥ 497.4	≥ 125	≥ 125	0.5415
PC-l-Leu-Me	≥ 125	≥ 497.4	≥ 125	≥ 125	0.5415
PC-MeAcr	62.5	301.7	≥ 125	62.5	−0.5555
**PC-l-Met-Me**	**< 3.91**	**< 14.5**	≥ 500	62.5	−0.2175
**PC-d/l-Pgl-Me**	**< 1.95**	**< 7.3**	≥ 250	**31.25**	0.5745
**PC-dl-Phe-Et**	**31.25**	**104.4**	≥ 125	62.5	1.0035
PC-d-Ser	≥ 250	≥ 1183.8	125	62.5	−2.0505
PC-d-Ser-Me	250	1110.1	≥ 500	250	−1.9075
PC-l-Ser-Me	≥ 500	≥ 2220.2	≥ 500	≥ 500	−1.9075
PC-l-*O*Bn-Ser	≥ 500	≥ 1659.5	250	62.5	0.3495
**PC-l-*O*Bn-Ser-Me**	**7.81**	**24.8**	≥ 500	62.5	0.4925
PC-d-*Ot*Bu-Ser-Me	≥ 500	≥ 1777.4	≥ 500	≥ 500	−0.0855
PC-l-*Ot*Bu-Ser-Me	≥ 500	≥ 1777.4	250	62.5	−0.0855
PC-d-Thr-Me	≥ 500	≥ 2090.0	≥ 500	≥ 500	−1.4455
PC-l-Thr-Me	≥ 500	≥ 2090.0	≥ 500	≥ 500	−1.4455
PC-d-*Ot*Bu-Thr-Me	62.5	211.6	≥ 500	≥ 500	0.3765
PC-l-*Ot*Bu-Thr-Me	≥ 500	≥ 1693.0	≥ 500	≥ 500	0.3765
PC-d-Trp-Et	62.5	184.7	250	62.5	1.3695
PC-l-Trp-Et	125	369.4	250	62.5	1.3695
PC-d-Tyr-Me	125	414.9	≥ 500	≥ 250	0.3545
**PC-l-Tyr-Et**	**7.81**	**24.8**	≥ 250	≥ 250	0.6955
PC-dl-Val-Et	≥ 125	≥ 497.4	≥ 125	≥ 125	0.4405
PC-d-Val-Me	≥ 125	≥ 526.9	≥ 125	≥ 125	0.0995
PC-l-Val-Me	≥ 125	≥ 526.9	≥ 125	≥ 125	0.0995
PZA	< 3.91	< 31.8	≥ 500	250	−1.4595
INH	0.125–0.25	0.91–1.82	7.81–15.625	3.91–7.81	−0.7970
RIF	0.0016–0.0062	0.0019–0.0075	6.25–12.5	0.39–1.56	4.5560
CPX	0.25	0.75	0.0625–0.25	0.0625	1.0370

* calculated from µg/mL, ** log*P*—calculated in MOE 2019.0101 (CCG, Quebec, Canada), PZA—pyrazinamide, INH—isoniazid, RIF—rifampicin, CPX—ciprofloxacin.

**Table 4:** Calculated selectivity index (SI) of the most active compounds against Mtb at pH 6 and pH 6.6. The compounds with a good cytotoxicity profile (SI > 10) are in bold.

Tested compound	Tested range (μM)	HepG2 IC_50_ (µM)	Mtb pH 6 MIC (µM)	SI pH 6
PC-l-Ala	1–1000	> 250*	≥ 1281	≥ 0.2
**PC-l-Ala-Et**	1–1000	> 1000	18	**> 57.1**
**PC-dl-Leu-Et**	1–1000	788.2	59	**13.4**
PC-MeAcr	1–500	704.2 **	302	1.7
PC-l-Met-Me	1–1000	> 100 *	15	6.9
**PC-d/l-Pgl-Me**	1–1000	> 1000	< 7	**> 68.5**
**PC-l-*O*** **Bn-Ser-Me**	1–1000	742.3	25	**30.0**
PC-l-*Ot*Bu-Thr-Me	1–500	> 500	1693	0.3
PC-l-Trp-Et	1–500	146.6	125	1.2
PC-l-Tyr-Et	1–1000	177.1	25	7.2
PZA		9619.1 ***	31.8	302.9

* The determination of IC_50_ was impossible due to irrelevant values of absorbance at higher concentrations of compounds caused by their precipitation in cell culture medium, ** the estimated value based on the curve, *** converted to µM based on the literature value 1184.3 ± 120.2 µg/mL [62].

## Supplementary Materials

The following are available online. Supplementary material (PDF) containing representative spectra, methodologies and results of antibacterial and antifungal screening, chiral HPLC chromatograms, description of calculated properties. SMILES, calculated properties and MIC against Mtb H37Ra of the title compounds (CSV).

## Abbreviations

aa—amino acid, CDI—1,1′-carbonyldiimidazole, DMAP—4-(dimethylamino)pyridine, EtOAc—ethyl acetate, MeAcr—methyl acrylate, MIC—minimum inhibitory concentration, MoA—mechanism of action, mp—melting point, Mtb—*Mycobacterium tuberculosis*, PC—pyrazinecarbonyl, Pgl—phenylglycine, POA—pyrazinoic acid, PZA—pyrazinamide, THF—tetrahydrofurane, TLC—thin-layer chromatography, TMSCl—trimethylsilyl chloride.

## References

[1] World Health Organization (2019). Global Tuberculosis Report 2019.

[2] von Reyn C.F., Waddell R.D., Eaton T., Arbeit R.D., Maslow J.N., Barber T.W., Brindle R.J., Gilks C.F., Lumio J., Lahdevirta J. (1993). Isolation of Mycobacterium Avium Complex from Water in the United States, Finland, Zaire, and Kenya. J. Clin. Microbiol..

[3] Tortoli E. (2003). Impact of Genotypic Studies on Mycobacterial Taxonomy: The New Mycobacteria of the 1990s. Clin. Microbiol. Rev..

[4] Johnson M.M., Odell J.A. (2014). Nontuberculous Mycobacterial Pulmonary Infections. J. Thorac. Dis..

[5] Yeager R.L., Munroe W.G., Dessau F.I. (1952). Pyrazinamide (Aldinamide) in the Treatment of Pulmonary Tuberculosis. Am. Rev. Tuberc..

[6] Zhang Y., Shi W., Zhang W., Mitchison D. (2014). Mechanisms of Pyrazinamide Action and Resistance. Microbiol. Spectr..

[7] Petrella S., Gelus-Ziental N., Maudry A., Laurans C., Boudjelloul R., Sougakoff W. (2011). Crystal Structure of the Pyrazinamidase of Mycobacterium Tuberculosis: Insights into Natural and Acquired Resistance to Pyrazinamide. PLOS ONE.

[8] Zitko J., Dolezal M. (2018). Old Drugs and New Targets as an Outlook for the Treatment of Tuberculosis. Curr. Med. Chem..

[9] Zimhony O., Vilcheze C., Arai M., Welch J.T., Jacobs W.R. (2007). Pyrazinoic Acid and Its N-Propyl Ester Inhibit Fatty Acid Synthase Type I in Replicating Tubercle Bacilli. Antimicrob. Agents Chemother..

[10] Zimhony O., Cox J.S., Welch J.T., Vilcheze C., Jacobs W.R. (2000). Pyrazinamide Inhibits the Eukaryotic-Like Fatty Acid Synthetase I (FASI) of Mycobacterium Tuberculosis. Nat. Med..

[11] Shi W., Zhang X., Jiang X., Yuan H., Lee J.S., Barry C.E., Wang H., Zhang W., Zhang Y. (2011). Pyrazinamide Inhibits Trans-Translation in Mycobacterium Tuberculosis. Science.

[12] Sun Q., Li X., Perez L.M., Shi W., Zhang Y., Sacchettini J.C. (2020). The Molecular Basis of Pyrazinamide Activity on Mycobacterium Tuberculosis Pand. Nat. Commun..

[13] Kim H., Shibayama K., Rimbara E., Mori S. (2014). Biochemical Characterization of Quinolinic Acid Phosphoribosyltransferase from Mycobacterium Tuberculosis H37rv and Inhibition of Its Activity by Pyrazinamide. PLOS ONE.

[14] He L., Cui P., Shi W., Li Q., Zhang W., Li M., Zhang Y. (2019). Pyrazinoic Acid Inhibits the Bifunctional Enzyme (Rv2783) in Mycobacterium Tuberculosis by Competing with Tmrna. Pathogens.

[15] Zhang S., Chen J., Shi W., Cui P., Zhang J., Cho S., Zhang W., Zhang Y. (2017). Mutation in Clpc1 Encoding an Atp-Dependent Atpase Involved in Protein Degradation Is Associated with Pyrazinamide Resistance in Mycobacterium Tuberculosis. Emerg. Microbes Infect..

[16] Sheen P., Requena D., Gushiken E., Gilman R.H., Antiparra R., Lucero B., Lizarraga P., Cieza B., Roncal E., Grandjean L. (2017). A Multiple Genome Analysis of Mycobacterium Tuberculosis Reveals Specific Novel Genes and Mutations Associated with Pyrazinamide Resistance. BMC Genomics.

[17] Njire M., Wang N., Wang B., Tan Y., Cai X., Liu Y., Mugweru J., Guo J., Hameed H.M.A., Tan S. (2017). Pyrazinoic Acid Inhibits a Bifunctional Enzyme in Mycobacterium Tuberculosis. Antimicrob Agents Chemother..

[18] Gopal P., Gruber G., Dartois V., Dick T. (2019). Pharmacological and Molecular Mechanisms Behind the Sterilizing Activity of Pyrazinamide. Trends Pharmacol. Sci..

[19] Via L.E., Savic R., Weiner D.M., Zimmerman M.D., Prideaux B., Irwin S.M., Lyon E., O’Brien P., Gopal P., Eum S. (2015). Host-Mediated Bioactivation of Pyrazinamide: Implications for Efficacy, Resistance, and Therapeutic Alternatives. ACS Infect. Dis..

[20] Correa M.F., Fernandes J.P. (2016). Pyrazinamide and Pyrazinoic Acid Derivatives Directed to Mycobacterial Enzymes against Tuberculosis. Curr. Protein. Pept. Sci..

[21] Cynamon M.H., Gimi R., Gyenes F., Sharpe C.A., Bergmann K.E., Han H.J., Gregor L.B., Rapolu R., Luciano G., Welch J.T. (1995). Pyrazinoic Acid Esters with Broad Spectrum in Vitro Antimycobacterial Activity. J. Med. Chem..

[22] Cynamon M.H., Klemens S.P., Chou T.S., Gimi R.H., Welch J.T. (1992). Antimycobacterial Activity of a Series of Pyrazinoic Acid Esters. J. Med. Chem..

[23] Bergmann K.E., Cynamon M.H., Welch J.T. (1996). Quantitative Structure-Activity Relationships for the in Vitro Antimycobacterial Activity of Pyrazinoic Acid Esters. J. Med. Chem..

[24] Simoes M.F., Valente E., Gomez M.J., Anes E., Constantino L. (2009). Lipophilic Pyrazinoic Acid Amide and Ester Prodrugs Stability, Activation and Activity against M. Tuberculosis. Eur. J. Pharm. Sci..

[25] Semelkova L., Jandourek O., Konecna K., Paterova P., Navratilova L., Trejtnar F., Kubicek V., Kunes J., Dolezal M., Zitko J. (2017). 3-Substituted N-Benzylpyrazine-2-Carboxamide Derivatives: Synthesis, Antimycobacterial and Antibacterial Evaluation. Molecules.

[26] Zitko J., Jand’ourek O., Paterova P., Navratilova L., Kunes J., Vinsova J., Dolezal M. (2018). Design, Synthesis and Antimycobacterial Activity of Hybrid Molecules Combining Pyrazinamide with a 4-Phenylthiazol-2-Amine Scaffold. Medchemcomm.

[27] Vale N., Ferreira A., Matos J., Fresco P., Gouveia M.J. (2018). Amino Acids in the Development of Prodrugs. Molecules.

[28] Ibrahim M.A., Panda S.S., Birs A.S., Serrano J.C., Gonzalez C.F., Alamry K.A., Katritzky A.R. (2014). Synthesis and Antibacterial Evaluation of Amino Acid-Antibiotic Conjugates. Bioorg. Med. Chem. Lett..

[29] Pochopin N.L., Charman W.N., Stella V.J. (1995). Amino-Acid Derivatives of Dapsone as Water-Soluble Prodrugs. Int. J. Pharm..

[30] Kushner S., Dalalian H., Sanjurjo J.L., Bach F.L., Safir S.R., Smith V.K., Williams J.H. (1952). Experimental Chemotherapy of Tuberculosis. Ii. The Synthesis of Pyrazinamides and Related Compounds1. J. Am. Chem. Soc..

[31] Badie M.F., Azab M.S. (1991). Synthesis and Antimicrobial Activity of Some Pyrazine- and Disubstituted Pyrazine-Amino Acid Derivatives. Alex. J. Pharm. Sci..

[32] Pinheiro A.C., Kaiser C.R., Lourenco M.C.S., de Souzaa M.V.N., Wardell S.M.S.V., Wardell J.L. (2007). Synthesis and in Vitro Activity Towards Mycobacterium Tuberculosis of l-Serinyl Ester and Amino Derivatives of Pyrazinoic Acid. Chemin-.

[33] Panda S.S., Girgis A.S., Mishra B.B., Elagawany M., Devarapalli V., Littlefield W.F., Samir A., Fayad W., Fawzy N.G., Srour A.M. (2019). Synthesis, Computational Studies, Antimycobacterial and Antibacterial Properties of Pyrazinoic Acid-Isoniazid Hybrid Conjugates. RSC Advances.

[34] Makino E., Iwasaki N., Yagi N., Ohashi T., Kato H., Ito Y., Azuma H. (1990). Studies on Antiallergic Agents. I. Synthesis and Antiallergic Activity of Novel Pyrazine Derivatives. Chem. Pharm. Bull. (Tokyo).

[35] Himaja M., Venkataramana M., Shaifali M., Kilaru J.P., Ranjitha A., Saisaraswathi V., Asif K. (2010). Ultrasound-Mediated Synthesis Pyrazine-2-Carboxylamino Acids and Dipeptides as Potent Insecticidal and Anthelmintic Agents. Int. J. Res. Ayurveda Pharm..

[36] Moreira W., Santhanakrishnan S., Ngan G.J.Y., Low C.B., Sangthongpitag K., Poulsen A., Dymock B.W., Dick T. (2017). Towards Selective Mycobacterial Clpp1p2 Inhibitors with Reduced Activity against the Human Proteasome. Antimicrob. Agents Chemother..

[37] Larsen E.M., Johnson R.J. (2019). Microbial Esterases and Ester Prodrugs: An Unlikely Marriage for Combating Antibiotic Resistance. Drug Dev. Res..

[38] Li J., Sha Y. (2008). A Convenient Synthesis of Amino Acid Methyl Esters. Molecules.

[39] Amblard M., Fehrentz J.A., Martinez J., Subra G. (2006). Methods and Protocols of Modern Solid Phase Peptide Synthesis. Mol. Biotechnol..

[40] Hill T.A., Lohman R.J., Hoang H.N., Nielsen D.S., Scully C.C., Kok W.M., Liu L., Lucke A.J., Stoermer M.J., Schroeder C.I. (2014). Cyclic Penta- and Hexaleucine Peptides without N-Methylation Are Orally Absorbed. ACS Med. Chem. Lett..

[41] Yamada S., Hongo C., Yoshioka R., Chibata I. (1983). Method for the Racemization of Optically-Active Amino-Acids. J. Org. Chem..

[42] Paul R., Anderson G.W. (1960). N,N‘-Carbonyldiimidazole, a New Peptide Forming Reagent. J. Am. Chem. Soc..

[43] E-Abadelah M.M., Sabri S.S., Jarrar A.A., Zarga M.H.A. (1979). Chiroptical Properties of N-(2-Pyrazinoyl)-A-Amino-Esters, -Aziridines, and Related Compounds. J. Chem. Soc. Perkin Trans. 1.

[44] Popovic S., Bieraugel H., Detz R.J., Kluwer A.M., Koole J.A., Streefkerk D.E., Hiemstra H., van Maarseveen J.H. (2013). Epimerization-Free C-Terminal Peptide Activation. Chemistry.

[45] Franzblau S.G., Witzig R.S., McLaughlin J.C., Torres P., Madico G., Hernandez A., Degnan M.T., Cook M.B., Quenzer V.K., Ferguson R.M. (1998). Rapid, Low-Technology Mic Determination with Clinical Mycobacterium Tuberculosis Isolates by Using the Microplate Alamar Blue Assay. J. Clin. Microbiol.

[46] Namouchi A., Cimino M., Favre-Rochex S., Charles P., Gicquel B. (2017). Phenotypic and Genomic Comparison of Mycobacterium Aurum and Surrogate Model Species to Mycobacterium Tuberculosis: Implications for Drug Discovery. BMC Genomics.

[47] Chaturvedi V., Dwivedi N., Tripathi R.P., Sinha S. (2007). Evaluation of Mycobacterium Smegmatis as a Possible Surrogate Screen for Selecting Molecules Active against Multi-Drug Resistant Mycobacterium Tuberculosis. J. Gen. Appl. Microbiol..

[48] Heinrichs M.T., May R.J., Heider F., Reimers T., SK B.S., Peloquin C.A., Derendorf H. (2018). Mycobacterium Tuberculosis Strains H37ra and H37rv Have Equivalent Minimum Inhibitory Concentrations to Most Antituberculosis Drugs. Int. J. Mycobacteriol..

[49] Yamamoto S., Toida I., Watanabe N., Ura T. (1995). In Vitro Antimycobacterial Activities of Pyrazinamide Analogs. Antimicrob. Agents Chemother..

[50] Vandal O.H., Nathan C.F., Ehrt S. (2009). Acid Resistance in Mycobacterium Tuberculosis. J. Bacteriol..

[51] den Hertog A.L., Menting S., Pfeltz R., Warns M., Siddiqi S.H., Anthony R.M. (2016). Pyrazinamide Is Active against Mycobacterium Tuberculosis Cultures at Neutral Ph and Low Temperature. Antimicrob. Agents Chemother..

[52] Bansa-Mutalik R., Nikaido H. (2014). Mycobacterial Outer Membrane Is a Lipid Bilayer and the Inner Membrane Is Unusually Rich in Diacyl Phosphatidylinositol Dimannosides. Proc. Natl. Acad. Sci. USA.

[53] Chen H., Nyantakyi S.A., Li M., Gopal P., Aziz D.B., Yang T., Moreira W., Gengenbacher M., Dick T., Go M.L. (2018). The Mycobacterial Membrane: A Novel Target Space for Anti-Tubercular Drugs. Front. Microbiol..

[54] (2019). Molecular Operating Environment (MOE).

[55] Palos I., Luna-Herrera J., Lara-Ramirez E.E., Loera-Piedra A., Fernandez-Ramirez E., Aguilera-Arreola M.G., Paz-Gonzalez A.D., Monge A., Wan B., Franzblau S. (2018). Anti-Mycobacterium Tuberculosis Activity of Esters of Quinoxaline 1,4-Di-N-Oxide. Molecules.

[56] Adachi H., Tsujimoto M. (1995). Cloning and Expression of Dipeptidase from Acinetobacter Calcoaceticus Atcc 23055. J. Biochem..

[57] Reichau S., Blackmore N.J., Jiao W., Parker E.J. (2016). Probing the Sophisticated Synergistic Allosteric Regulation of Aromatic Amino Acid Biosynthesis in Mycobacterium Tuberculosis Using -Amino Acids. PLOS ONE.

[58] European Committee for Antimicrobial Susceptibility Testing (Eucast) of the European Society for Clinical Microbiology and Infectious Diseases (Escmid) (2003). Eucast Discussion Document E. Dis 5.1: Determination of Minimum Inhibitory Concentrations (Mics) of Antibacterial Agents by Broth Dilution. Clin. Microbiol. Infec..

[59] (2017). Eucast Definitive Document E.Def 7.3.1. Method for the Determination of Broth Dilution Minimum Inhibitory Concentrations of Antifungal Agents for Yeasts. http://www.eucast.org/astoffungi/methodsinantifungalsusceptibilitytesting/susceptibility_testing_of_yeasts/.

[60] (2017). Eucast Definitive Document E.Def 9.3.1. Method for the Determination of Broth Dilution Minimum Inhibitory Concentrations of Antifungal Agents for Conidia Forming Moulds. http://www.eucast.org/astoffungi/methodsinantifungalsusceptibilitytesting/susceptibility_testing_of_moulds/.

[61] Bagla V.P., McGaw L.J., Elgorashi E.E., Eloff J.N. (2014). Antimicrobial Activity, Toxicity and Selectivity Index of Two Biflavonoids and a Flavone Isolated from Podocarpus Henkelii (Podocarpaceae) Leaves. BMC Complement. Altern. Med..

[62] Shih T.Y., Pai C.Y., Yang P., Chang W.L., Wang N.C., Hu O.Y. (2013). A Novel Mechanism Underlies the Hepatotoxicity of Pyrazinamide. Antimicrob. Agents Chemother..

[63] Kočevar M., Polanc S., Verček B., Tišler M. (1988). Syntheses of Some N-(Pyrazinecarbonyl) Amino Acids and Peptides. Recl. Trav. Chim. Pays.-Bas.

[64] Kakemi K., Arta T., Kitazawa S., Kiyotaki T. (1961). Studies on the Synthesis of Pyrazinoic Acid Derivatives. Ii. Derivatives of 3-Aminopyrazinoic Acid. Yakugaku Zasshi.

[65] Naredla R.R., Dash B.P., Klumpp D.A. (2013). Preparation of Pyrazine Carboxamides: A Reaction Involving N-Heterocyclic Carbene (Nhc) Intermediates. Org. Lett..

[66] Cynamon M.H., Speirs R.J., Welch J.T. (1998). In Vitro Antimycobacterial Activity of 5-Chloropyrazinamide. Antimicrob. Agents Chemother..

[67] Fieweger R.A., Wilburn K.M., VanderVen B.C. (2019). Comparing the Metabolic Capabilities of Bacteria in the Mycobacterium Tuberculosis Complex. Microorganisms.

[68] Agapova A., Serafini A., Petridis M., Hunt D.M., Garza-Garcia A., Sohaskey C.D., de Carvalho L.P.S. (2019). Flexible Nitrogen Utilisation by the Metabolic Generalist Pathogen Mycobacterium Tuberculosis. eLife.

[69] Naffin-Olivos J.L., Daab A., White A., Goldfarb N.E., Milne A.C., Liu D., Baikovitz J., Dunn B.M., Rengarajan J., Petsko G.A. (2017). Structure Determination of Mycobacterium Tuberculosis Serine Protease Hip1 (Rv2224c). Biochemistry.

[70] Akopian T., Kandror O., Tsu C., Lai J.H., Wu W., Liu Y., Zhao P., Park A., Wolf L., Dick L.R. (2015). Cleavage Specificity of Mycobacterium Tuberculosis Clpp1p2 Protease and Identification of Novel Peptide Substrates and Boronate Inhibitors with Anti-Bacterial Activity. J. Biol. Chem..

[71] Soni D.K., Dubey S.K., Bhatnagar R. (2020). Atp-Binding Cassette (Abc) Import Systems of Mycobacterium Tuberculosis: Target for Drug and Vaccine Development. Emerg. Microbes Infect..

[72] Dasgupta A., Sureka K., Mitra D., Saha B., Sanyal S., Das A.K., Chakrabarti P., Jackson M., Gicquel B., Kundu M. (2010). An Oligopeptide Transporter of Mycobacterium Tuberculosis Regulates Cytokine Release and Apoptosis of Infected Macrophages. PLOS ONE.

