# Supplementary material for: N-Pyrazinoyl Substituted Amino Acids as Potential Antimycobacterial Agents—the Synthesis and Biological Evaluation of Enantiomers
Source: Molecules. 2020 Mar 27;25(7):1518. doi: 10.3390/molecules25071518 (PMC7181131; doi:10.3390/molecules25071518)
Supplement: Supplementary file 1 [file molecules-25-01518-s001.zip › Supplementary Material.pdf]

Supplementary Material

N-pyrazinoyl substituted amino acids as potential antimycobacterial agents – synthesis and biological evaluation of enantiomers

Martin Juhás [1,*], Lucie Kučerová [1], Ondřej Horáček [1], Ondřej Janďourek [1], Vladimír Kubíček [1], Klára Konečná [1], Radim Kučera [1], Pavel Bárta [1], Jiří Janoušek [1], Pavla Paterová [2], Jiří Kuneš [1], Martin Doležal [1] and Jan Zitko [1,*]

[1] Charles University, Faculty of Pharmacy in Hradec Králové, Akademika Heyrovského 1203, Hradec Králové, Czech Republic; juhasm@faf.cuni.cz
[2] University Hospital Hradec Králové, Department of Clinical Microbiology, Sokolská 581, 500 05 Hradec Králové, Czech Republic
* Correspondence: juhasm@faf.cuni.cz (M.J.); jan.zitko@faf.cuni.cz (J.Z.); Tel.: +420-495-067-272 (M.J.); +420-495-067-409 (J.Z.)

Table of Contents

1. NMR spectra of representative compounds 2
   PC-L-Ala 2
   PC-L-Val-Me 3
   PC-D/L-Pgl-Me 4
   PC-DL-Phe-Et 5
2. Chiral HPLC chromatograms 6
3. In vitro antimycobacterial activity screening on Mtb H37Rv, M. kansasii and M. avium 9
   3.1. Methodology 9
   3.2. Results 9
4. List of abbreviations of tested strains in antibacterial and antifungal screening 10
5. In vitro antibacterial activity screening 11
   5.1. Methodology 11
   5.2. Internal standards 12
   5.3. Results 13
6. In vitro antifungal activity screening 14
   6.1. Methodology 14
   6.2. Internal standards 15
   6.3. Results 16
7. Description of the supplied csv file 17
8. References 18

1. NMR spectra of representative compounds

PC-L-Ala

1H-NMR

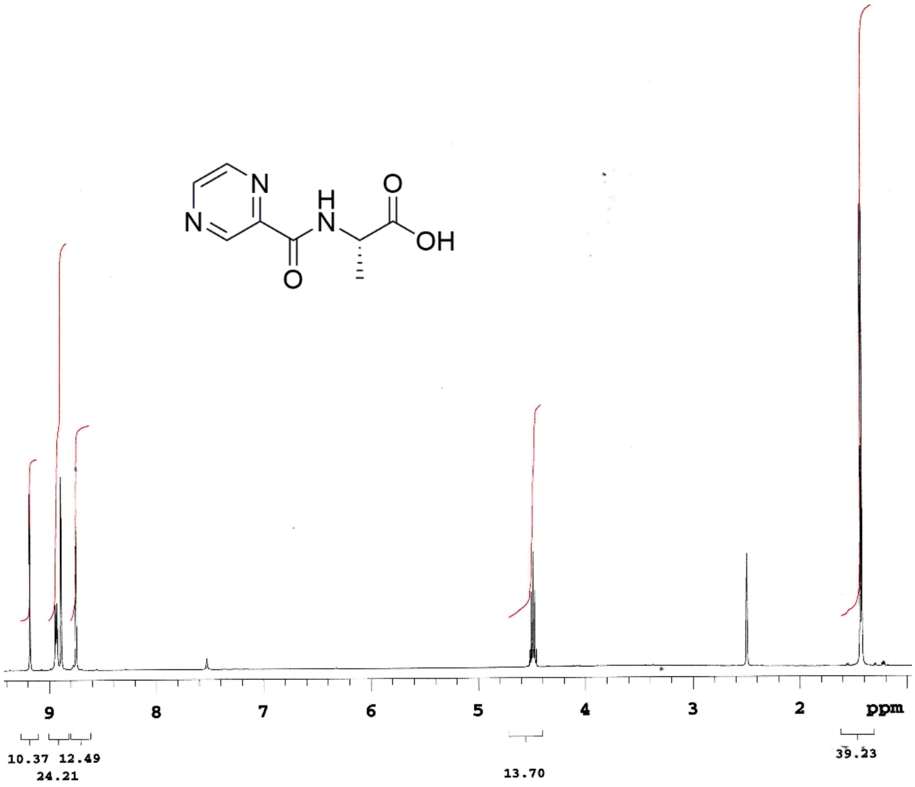

13C-NMR

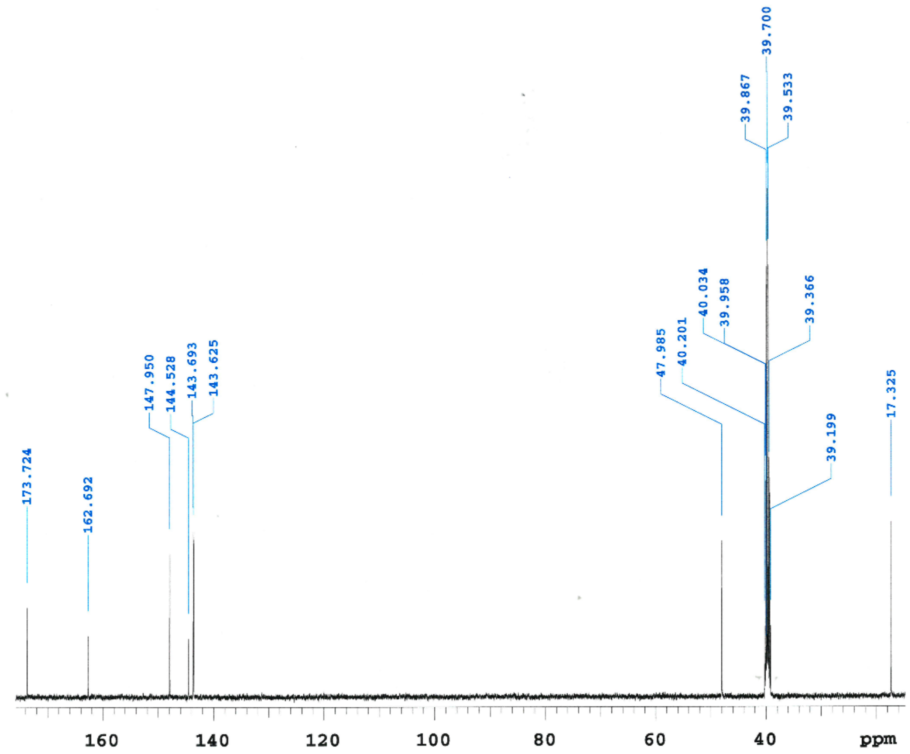

PC-L-Val-Me

^{1}H-NMR

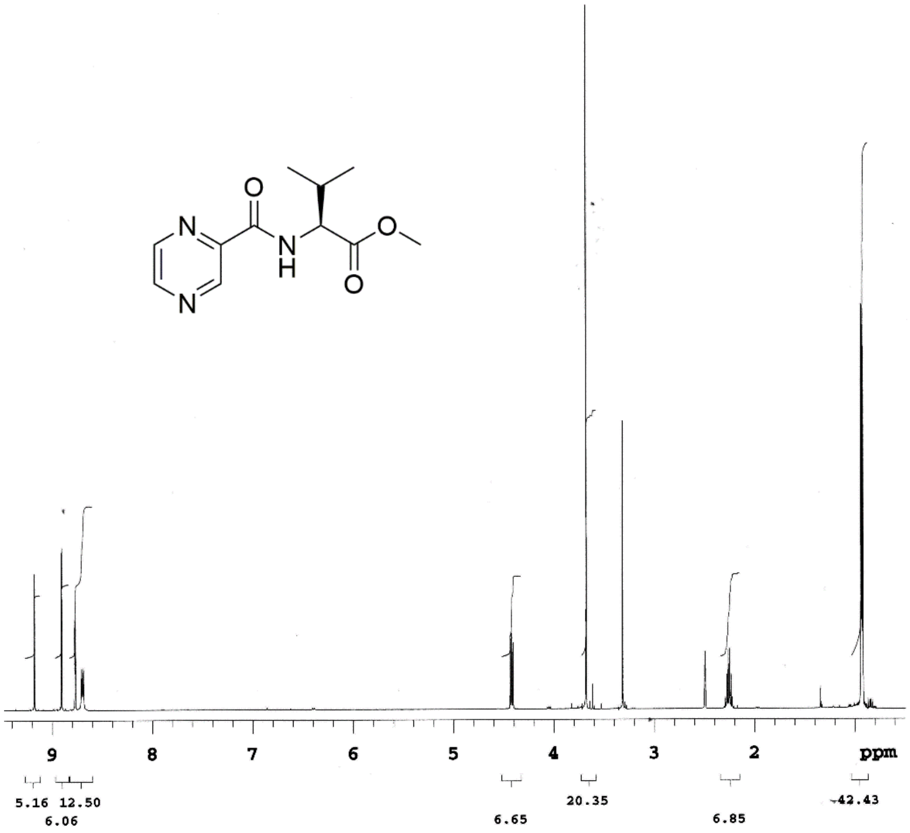

^{13}C-NMR

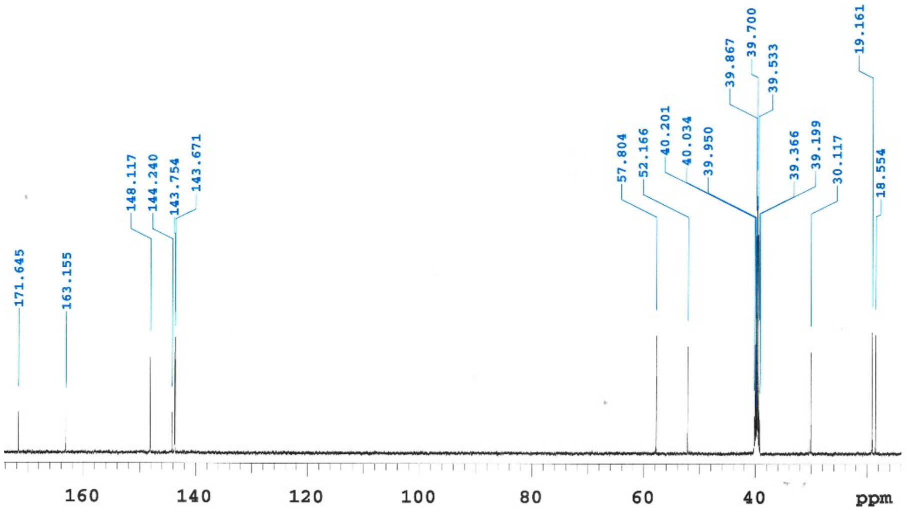

PC-D/L-Pgl-Me

^{1}H-NMR

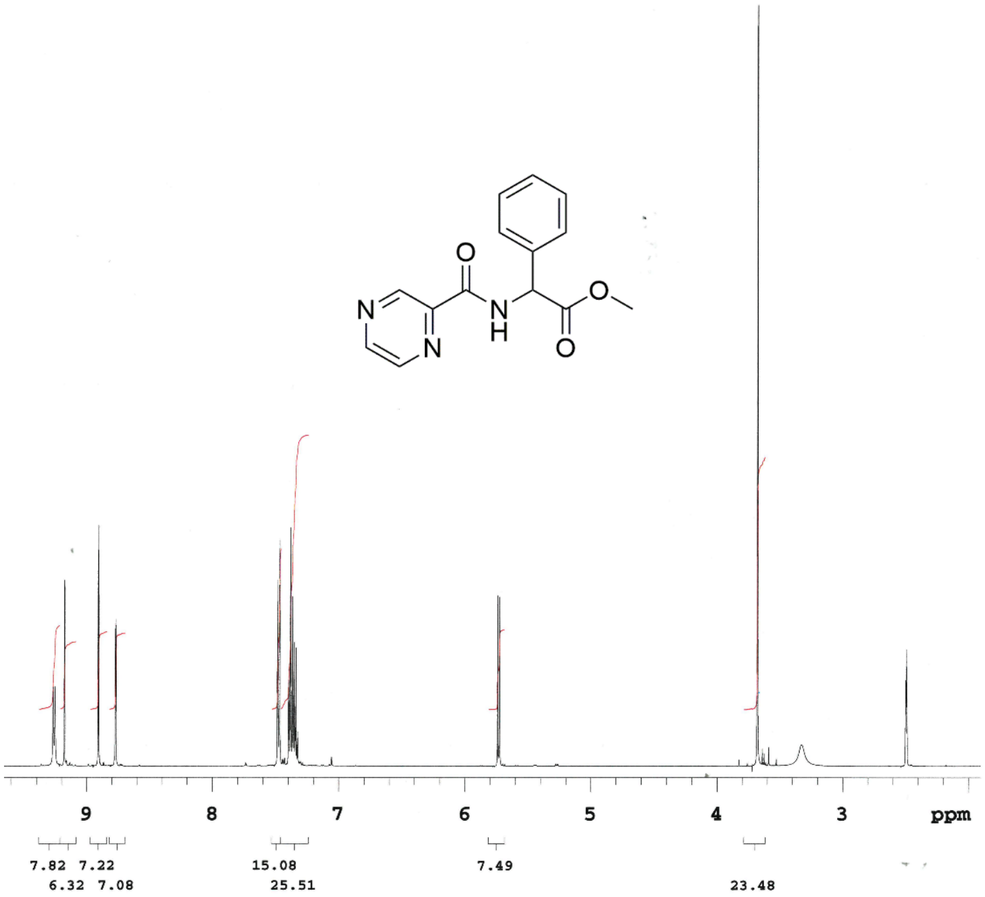

^{13}C-NMR

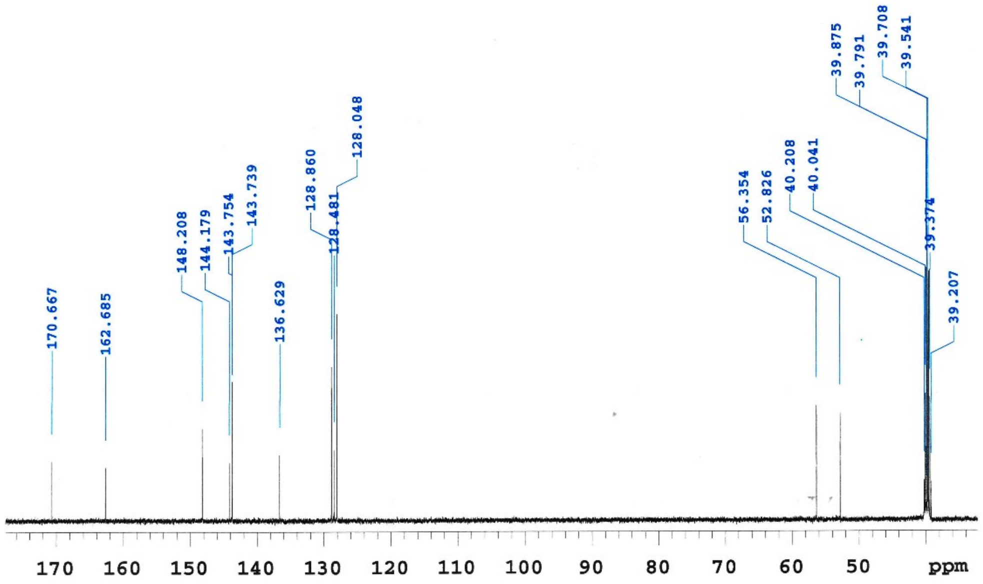

PC-*DL*-Phe-Et

^{1}H-NMR

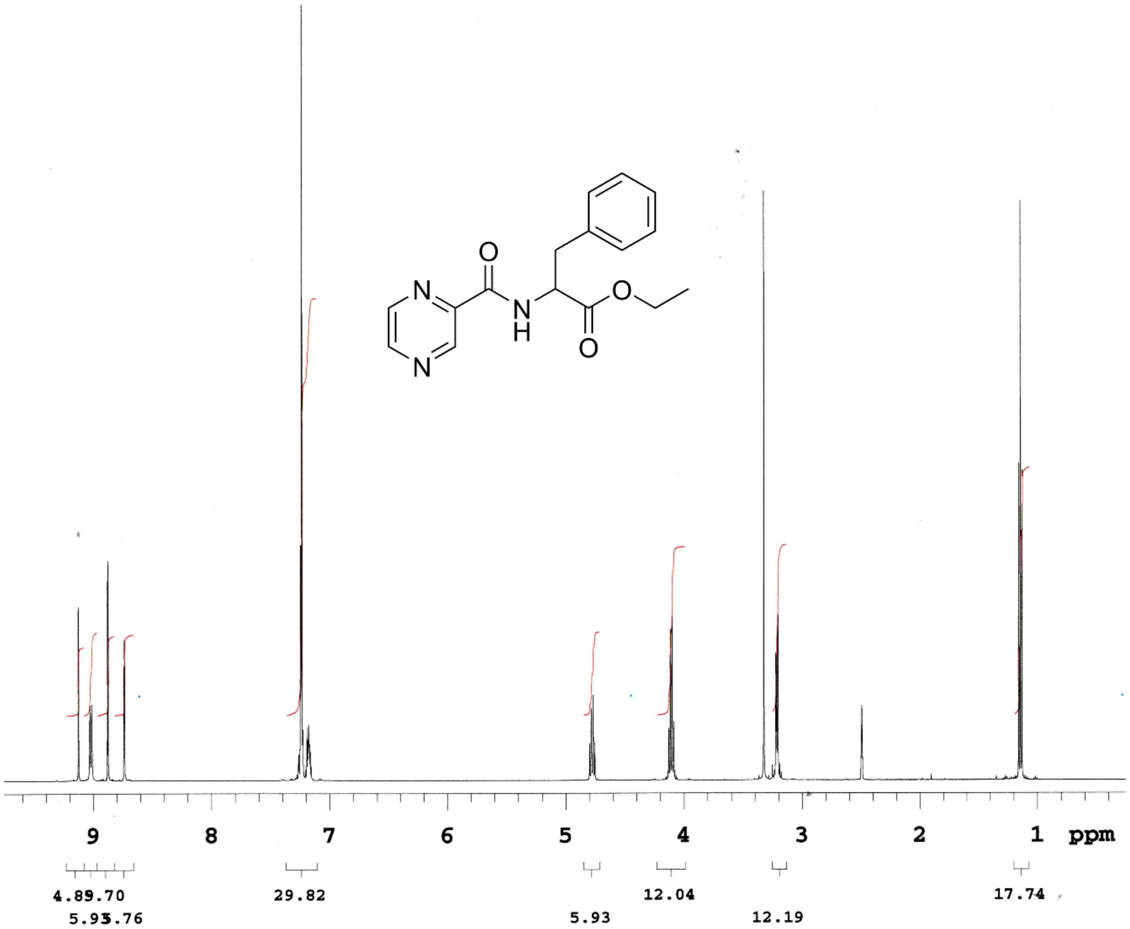

^{13}C-NMR

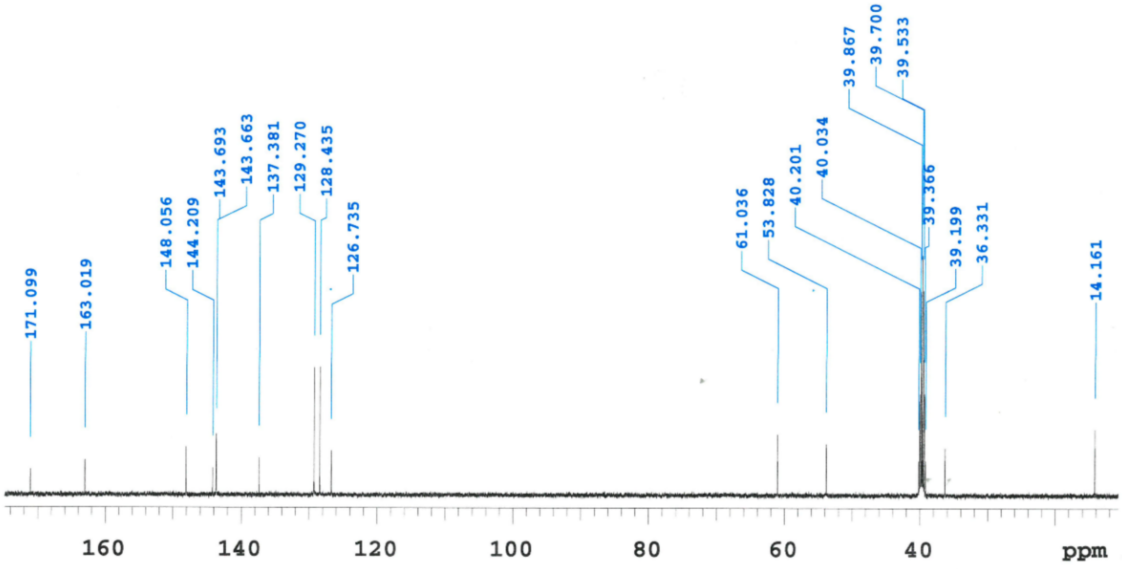

2. Chiral HPLC chromatograms

Table S1. Conditions used for chromatographic separation.

Analyzed Sample(s)	Used conditions
PC-D,L-Ala	methanol – 0.2% acetic acid (60/40; v/v)
PC-D,L-Ala-Et	acetonitrile – 50 mM aqueous $NaClO_4$ (45/55; v/v)
PC-D,L-Ile-Me	acetonitrile – 50 mM aqueous $NaClO_4$ (45/55; v/v)
PC-D,L-Leu-Me	acetonitrile – 50 mM aqueous $NaClO_4$ (45/55; v/v)
PC-D,L-Ser-Me	acetonitrile – 50 mM aqueous $NaClO_4$ (45/55; v/v)
PC-D,L-*Ot*Bu-Ser-Me	acetonitrile – 50 mM aqueous $NaClO_4$ (35/65; v/v)
PC-D,L-Thr-Me	acetonitrile – 50 mM aqueous $NaClO_4$ (45/55; v/v)
PC-D,L-*Ot*Bu-Thr-Me	acetonitrile – 50 mM aqueous $NaClO_4$ (45/55; v/v)
PC-D,L-Trp-Et	acetonitrile – 50 mM aqueous $NaClO_4$ (45/55; v/v)
PC-D,L-Val-Me	methanol – 50 mM aqueous $NaClO_4$ (60/40; v/v)
PC-DL-Ala-Me	acetonitrile – 50 mM aqueous $NaClO_4$ (45/55; v/v)
PC-DL-Leu-Et	acetonitrile – 50 mM aqueous $NaClO_4$ (45/55; v/v)
PC-DL-Val-Et	acetonitrile – 50 mM aqueous $NaClO_4$ (45/55; v/v)
PC-D/L-PgI-Me	acetonitrile – 50 mM aqueous $NaClO_4$ (35/65; v/v)
PC-DL-Phe-Et	acetonitrile – 50 mM aqueous $NaClO_4$ (45/55; v/v)

D,L – denotes sample of D-isomer (A), sample of L-isomer (B) and artificial mix of A+B

DL – denotes one sample of racemic mixture

D/L – denotes one sample of mixture of enantiomers

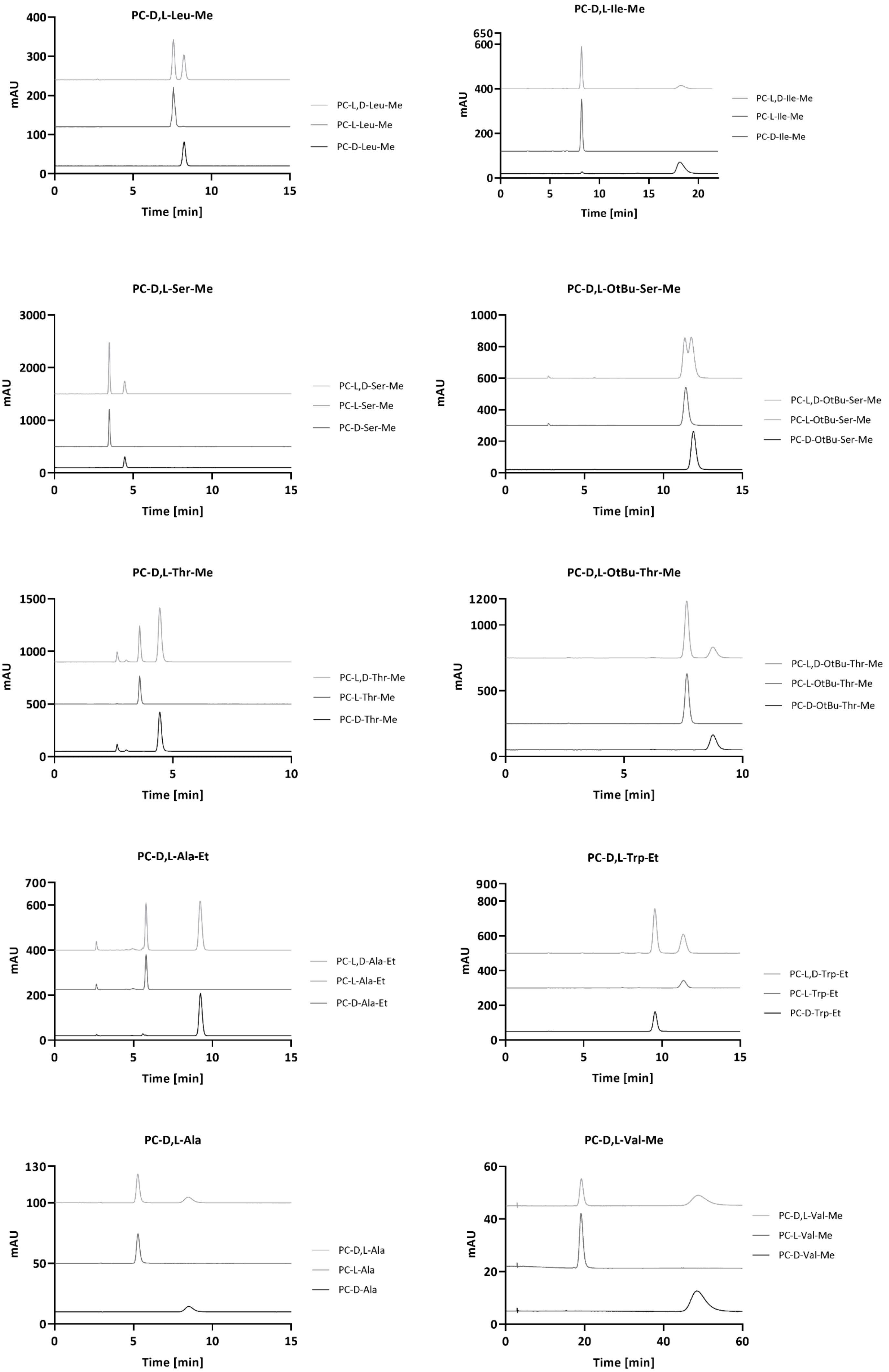

PC-D,L-Leu-Me
PC-L,D-Leu-Me
PC-L-Leu-Me
PC-D-Leu-Me
PC-D,L-Ile-Me
PC-L,D-Ile-Me
PC-L-Ile-Me
PC-D-Ile-Me
PC-D,L-Ser-Me
PC-L,D-Ser-Me
PC-L-Ser-Me
PC-D-Ser-Me
PC-D,L-OtBu-Ser-Me
PC-L,D-OtBu-Ser-Me
PC-L-OtBu-Ser-Me
PC-D-OtBu-Ser-Me
PC-D,L-Thr-Me
PC-L,D-Thr-Me
PC-L-Thr-Me
PC-D-Thr-Me
PC-D,L-OtBu-Thr-Me
PC-L,D-OtBu-Thr-Me
PC-L-OtBu-Thr-Me
PC-D-OtBu-Thr-Me
PC-D,L-Ala-Et
PC-L,D-Ala-Et
PC-L-Ala-Et
PC-D-Ala-Et
PC-D,L-Trp-Et
PC-L,D-Trp-Et
PC-L-Trp-Et
PC-D-Trp-Et
PC-D,L-Ala
PC-D,L-Ala
PC-L-Ala
PC-D-Ala
PC-D,L-Val-Me
PC-D,L-Val-Me
PC-L-Val-Me
PC-D-Val-Me
mAU
Time [min]

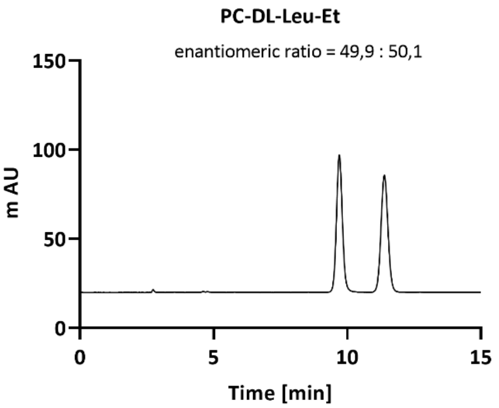
PC-DL-Leu-Et
enantiomeric ratio = 49,9 : 50,1
m AU
Time [min]

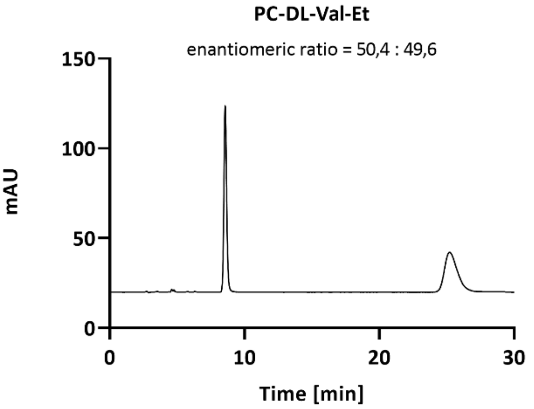
PC-DL-Val-Et
enantiomeric ratio = 50,4 : 49,6
mAU
Time [min]

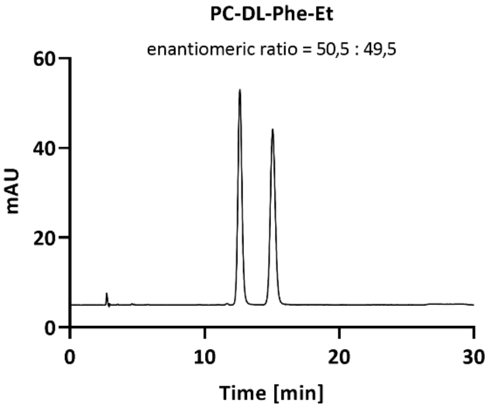
PC-DL-Phe-Et
enantiomeric ratio = 50,5 : 49,5
mAU
Time [min]

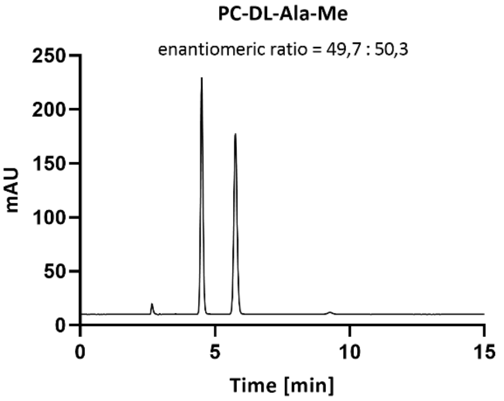
PC-DL-Ala-Me
enantiomeric ratio = 49,7 : 50,3
mAU
Time [min]

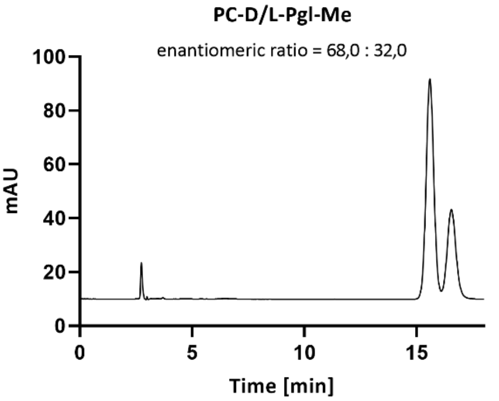
PC-D/L-Pgl-Me
enantiomeric ratio = 68,0 : 32,0
mAU
Time [min]

3. In vitro antimycobacterial activity screening on Mtb H37Rv, M. kansasii and M. avium

3.1. Methodology

A 96-well plate microdilution broth method was performed[1]. Tested strains *Mycobacterium tuberculosis* H37Rv CNCTC My 331/88 (ATCC 27294), *M. kansasii* CNCTC My 235/80 (ATCC 12478) and *M. avium* ssp. *avium* CNCTC My 80/72 (ATCC 15769) were obtained from the Czech National Collection of Type Cultures (CNCTC), National Institute of Public Health (Prague, Czech Republic). Middlebrook 7H9 broth of declared pH 6.6 (Sigma-Aldrich) enriched with 0.4% of glycerol (Sigma-Aldrich) and 10% of OADC growth supplement (oleic acid, albumin, dextrose, catalase; Himedia, Mumbai, India) was used for cultivation. Tested compounds were dissolved and diluted in DMSO and mixed with broth (25 µL of DMSO solution in 4.475 mL of broth) and placed (100 µL) into microplate wells. Mycobacterial inocula were suspended in isotonic saline solution and the density was adjusted to 0.5–1.0 according to McFarland scale. These suspensions were diluted by 10^{-1} and used to inoculate the testing wells, adding 100 µL of mycobacterial suspension per well. Final concentrations of tested compounds in wells were 100, 50, 25, 12.5, 6.25, 3.13 and 1.56 µg/mL. INH was used as positive control (inhibition of growth). Negative control (visible growth) consisted of broth plus mycobacterial suspension plus DMSO. A total of 30 µL of Alamar Blue working solution (1:1 mixture of 0.01% resazurin sodium salt (aq. sol.) and 10% Tween 80) was added after five days of incubation. Results were then determined after 24 h of incubation. The MIC (in µg/mL) was determined as the lowest concentration that prevented the blue to pink colour change. MIC values of INH are presented in Table S2. All experiments were conducted in duplicates.

3.2. Results

Table S2. Results of antimycobacterial screening on Mtb H37Rv, *M. kansasii* and *M. avium*. Results expressed as MIC in µg/mL.

Compound	Mtb H37Rv	*M. kansasii*	*M. avium*
PC-D-Ala	>100	>100	>100
PC-Gly	>100	>100	>100
PC-DL-Leu-Et	**100**	>100	>100
PC-D-Leu-Me	>100	>100	>100
PC-L-Leu-Me	100	>100	>100
PC-MeAcr	**50**	**100**	>100
PC-DL-Phe-Et	>100	>100	>100
PC-D-Ser	>100	>100	>100
PC-DL-Val-Et	>100	>100	>100
INH	0.39	25	25

4. List of abbreviations of tested strains in antibacterial and antifungal screening

Abbreviation	Full name
SA	*Staphylococcus aureus* subsp. *aureus* ATCC 29213, CCM 4223
MRSA	*Staphylococcus aureus* subsp. *aureus* MRSA, ATCC 43300, CCM 4750
SE	*Staphylococcus epidermidis*, ATCC 12228, CCM 4418
EF	*Enterococcus faecalis*, ATCC 29212, CCM 4224
EC	*Escherichia coli*, ATCC 25922, CCM 3954
KP	*Klebsiella pneumoniae*, ATCC 10031, CCM 4415
ACI	*Acinetobacter baumannii*, ATCC 19606, DSM 30007
PA	*Pseudomonas aeruginosa*, ATCC 27853, CCM 3955
CA	*Candida albicans* ATCC 24433, CCM 8320
CK	*Candida krusei* ATCC 6258, CCM 8271
CP	*Candida parapsilosis* ATCC 22019, CCM 8260
CT	*Candida tropicalis* ATCC 750, CCM 8264
AF	*Aspergillus fumigatus* ATCC 204305
AFla	*Aspergillus flavus* CCM 8363
LC	*Lichtheimia corymbifera* CCM 8077
TI	*Trichophyton interdigitale* ATCC 9533, CCM 8377

5. In vitro antibacterial activity screening

5.1. Methodology

The microdilution broth method was performed according to EUCAST (The European Committee on Antimicrobial Susceptibility Testing) instructions with slight modifications.[2] Eight tested bacterial strains were purchased from the Czech Collection of Microorganisms (CCM, Brno, Czech Republic) or from the German Collection of Microorganisms and Cell Cultures (DSM, Braunschweig, Germany): *Staphylococcus aureus* subsp. *aureus* CCM 4223 (ATCC 29213), *Staphylococcus aureus* subsp. *aureus* methicillin-resistant (MRSA) CCM 4750 (ATCC 43300), *Staphylococcus epidermidis* CCM 4418 (ATCC 12228), *Enterococcus faecalis* CCM 4224 (ATCC 29212), *Escherichia coli* CCM 3954 (ATCC 25922), *Klebsiella pneumoniae* CCM 4415 (ATCC 10031), *Acinetobacter baumannii* DSM 30007, ATCC 19606, *Pseudomonas aeruginosa* CCM 3955 (ATCC 27853). The cultivation was done in Cation-adjusted Mueller-Hinton broth (CAMHB, M-H 2 Broth, Sigma-Aldrich) at 35±2 °C. Tested compounds were dissolved in DMSO (Sigma-Aldrich, USA) to produce stock solutions. The final concentration of DMSO in the cultivation medium did not exceed 1% (v/v) of the total solution composition and did not affect the growth of bacteria. Positive controls consisted of test microbe solely, while negative controls consisted of cultivation medium and DMSO. Antibacterial activity was expressed as minimum inhibitory concentration (MIC, in μM) after 24 and 48 h of static incubation in the dark and humidified atmosphere, at 35±2 °C. Visual inspection and metabolic activity indicator, Alamar Blue (AlamarBlue™ Cell Viability reagent, ThermoFisher Scientific, USA), were used for MIC endpoint evaluation. The internal quality standards gentamycin and ciprofloxacin (both from Sigma-Aldrich) were involved in assays (for MIC of standards see Table S3). All experiments were conducted in duplicates. For the results to be valid, the difference in MIC for one compound determined from two parallel measurements must not be greater than one step on the dilution scale.

5.2. Internal standards

Table S3. Results of internal quality controls (standards) in antibacterial screening.

**Internal quality control**	**ciprofloxacin (mg/L)**		**gentamicin (mg/L)**	
**Bacterial strain**	**Spectrophotometric detection***	**visual detection****	**spectrophotometric detection***	**visual detection****
Staphylococcus aureus subsp. *aureus,* ATCC 29213, CCM 4223	0.256	0.256	1	0,5
Staphylococcus aureus subsp. *aureus,* MRSA, ATCC 43300, CCM 4750	0.128	0.128	>8	>8
Staphylococcus epidermidis ATCC 12228, CCM 4418	0.256	0.128	0.125	0.125
Enterococcus faecalis, ATCC 29212, CCM 4224	0.512	1.024	>8	>8
Escherichia coli, ATCC 25922, CCM 3954	0.008	0.008	1	1
Klebsiella pneumoniae, ATCC 10031, CCM 4415	0.008	0.008	0.5	0.5
Acinetobacter baumannii, ATCC 19606, DSM 30007	0.512	0.256	8	8
Pseudomonas aeruginosa, ATCC 27853, CCM 3955	0.512	0.512	0.5	0.5

Notes: Spectrophotometric detection- results were read with a microdilution plate reader (Synergy™ HTX, BioTek Instruments, Inc., USA) at wavelength 530 nm.

*The MIC of antibacterial agents are the lowest concentration giving rise to an inhibition of growth of 95% of that of the drug-free control. Results were read after 24 h microdilution plates cultivation without agitation at 35±2 °C in humidified atmosphere.

**The MIC was determined by naked eye in the well with the lowest drug concentration, where no visible growth of microbial agent was detected. Results were read after 24 h (bacteria, yeasts) or 48 h (moulds) microdilution plates cultivation without agitation at 35±2 °C in humidified atmosphere.

5.3. Results

Table S4. Results of in vitro antibacterial screening. For abbreviations see section 4. Results expressed as MIC in μM read after 24 h of incubation.

Compound	SA	MRSA	SE	EF	EC	KP	ACI	PA
PC-D-Ala	>500	>500	>500	>500	>500	>500	>500	>500
PC-L-Ala	>500	>500	>500	>500	>500	>500	>500	>500
PC-DL-Ala-Me	>500	>500	>500	>500	>500	>500	>500	>500
PC-D-Ala-Et	>500	>500	>500	>500	>500	>500	>500	>500
PC-L-Ala-Et	125	250	250	>500	>500	>500	>500	>500
PC-D-Asp-diMe	NA	NA	NA	NA	NA	NA	NA	NA
PC-L-Asp-diEt	>500	>500	500	>500	>500	>500	>500	>500
PC-L-*S*Bn-Cys	250	>250	250	>250	>250	>250	>250	>250
PC-D-Glu-diMe	>500	>500	>500	>500	>500	>500	>500	>500
PC-L-Glu-diEt	>500	>500	>500	>500	>500	>500	>500	>500
PC-Gly	>500	>500	>500	>500	>500	>500	>500	>500
PC-Gly-Et	>500	>500	>500	>500	>500	>500	>500	>500
PC-D-Ile-Me	NA	NA	NA	NA	NA	NA	NA	NA
PC-L-Ile-Me	NA	NA	NA	NA	NA	NA	NA	NA
PC-DL-Leu-Et	>500	>500	>500	>500	>500	>500	>500	>500
PC-D-Leu-Me	>500	>500	>500	>500	>500	>500	>500	>500
PC-L-Leu-Me	>500	>500	500	>500	>500	>500	>500	>500
PC-MeAcr	>125	>125	>125	>125	>125	>125	>125	>125
PC-L-Met-Me	>500	>500	>500	>500	>500	>500	>500	>500
PC-D/L-Pgl-Me	>500	>500	>500	>500	>500	>500	>500	>500
PC-DL-Phe-Et	>500	>500	500	>500	>500	>500	>500	>500
PC-D-Ser	>500	>500	>500	>500	>500	>500	>500	>500
PC-D-Ser-Me	250	>500	>500	>500	>500	>500	>500	>500
PC-L-Ser-Me	>500	>500	>500	>500	>500	>500	>500	>500
PC-L-*O*Bn-Ser	>500	>500	>500	>500	>500	>500	>500	>500
PC-L-*O*Bn-Ser-Me	>250	>250	>250	>250	>250	>250	>250	>250
PC-D-*Ot*Bu-Ser-Me	>500	>500	>500	>500	>500	>500	>500	>500
PC-L-*Ot*Bu-Ser-Me	>500	>500	>500	>500	>500	>500	>500	>500
PC-D-Thr-Me	>500	>500	>500	>500	>500	>500	>500	>500
PC-L-Thr-Me	500	500	>500	>500	>500	>500	>500	>500
PC-D-*Ot*Bu-Thr-Me	>500	>500	>500	>500	>500	>500	>500	>500
PC-L-*Ot*Bu-Thr-Me	**31.25**	**31.25**	62.5	>500	>500	>500	>500	>500
PC-D-Trp-Et	>500	>500	>500	>500	>500	>500	>500	>500
PC-L-Trp-Et	>500	>500	>500	>500	>500	>500	>500	>500
PC-D-Tyr-Me	>500	>500	>500	>500	>500	>500	>500	>500
PC-L-Tyr-Et	>500	>500	>500	>500	>500	>500	>500	>500
PC-DL-Val-Et	>500	>500	>500	>500	>500	>500	>500	>500
PC-D-Val-Me	>500	>500	>500	>500	>500	>500	>500	>500
PC-L-Val-Me	>500	>500	>500	>500	>500	>500	>500	>500

NA – results not available due to insolubility in the medium

6. In vitro antifungal activity screening

6.1. Methodology

Antifungal activity evaluation was performed using a microdilution broth method according to EUCAST instructions (The European Committee on Antimicrobial Susceptibility Testing) with slight modifications[3,4]. Eight fungal strains (four yeast and four mould strains) were used for antifungal activity screening, namely: *Candida albicans* CCM 8320 (ATCC 24433), *Candida krusei* CCM 8271 (ATCC 6258), *Candida parapsilosis* CCM 8260 (ATCC 22019), *Candida tropicalis* CCM 8264 (ATCC 750), *Aspergillus fumigatus* ATCC 204305, *Aspergillus flavus* CCM 8363, *Lichtheimia corymbifera* CCM 8077, and *Trichophyton interdigitale* CCM 8377 (ATCC 9533). Tested strains were purchased from the Czech Collection of Microorganisms (CCM, Brno, Czech Republic) or from the American Type Collection Cultures (ATCC, Manassas, VA, USA). Tested compounds were dissolved in DMSO and diluted in a twofold manner with RPMI 1640 medium, with glutamine and 2% glucose, buffered to pH 7.0 with MOPS (3-morpholinopropane-1-sulfonic acid). The final concentration of DMSO in the testing medium did not exceed 1% (v/v) of the total solution composition. Static incubation was performed in the dark and in humid atmosphere, at 35±2 °C, for 24 and 48 h (72 and 120 h for *Trichophyton interdigitale* respectively). Positive controls consisted of test microbe solely, while negative controls consisted of cultivation medium and DMSO. Visual inspection and metabolic activity indicator, Alamar Blue (ThermoFisher Scientific), were used for MIC endpoint evaluation. The internal quality standards, amphotericin B (Sigma-Aldrich) and voriconazole (Toronto research Chemicals, CA) were involved in assays (for MIC of standards see Table S5). All experiments were conducted in duplicates. For the results to be valid, the difference in MIC for one compound determined from two parallel measurements must not be greater than one step on the dilution scale.

6.2. Internal standards

Table S5. Results of internal quality controls (standards) in antifungal screening.

**Internal quality control**	**amphotericin B (mg/L)**		**voriconazole (mg/L)**	
**Yeast/mould strain**	**spectrophotometric detection***	**visual detection****	**spectrophotometric detection*****	**visual detection****
Candida albicans ATCC 24433, CCM 8320	0.5	0.5	0.03	>16
Candida krusei ATCC 6258, CCM 8271	0.5	0.5	0.25	>16
Candida parapsilosis ATCC 22019, CCM 8260	0.5	0.5	0.03	>16
Candida tropicalis ATCC 750, CCM 8264	1	1	0.0625	>16
Aspergillus fumigatus ATCC 204305	1	1	0.25	1
Aspergillus flavus CCM 8363	4	4	1	>16
Lichtheimia corymbifera CCM 8077	0.125	0.125	>16	>16
Trichophyton interdigitale ATCC 9533, CCM 8377	1	1	>16	>16

Notes: Spectrophotometric detection results were read with a microdilution plate reader (Synergy™ HTX) at wavelength 530 nm.

*The MIC of amphotericin B is the lowest concentration giving rise to an inhibition of growth of 90% of that of the drug-free control. Results were read after 24 h (yeasts) or 48 h (moulds) microdilution plates cultivation without agitation at t 35±2 °C in humidified atmosphere.

**The MIC was determined by naked eye in the well with the lowest drug concentration, where no visible growth of microbial agent was detected. Results were read after 24 h (bacteria, yeasts) or 48 h (moulds) microdilution plates cultivation without agitation at 35±2 °C in humidified atmosphere.

****The MIC of azole (voriconazole) antifungal agents is the lowest drug concentration giving inhibition of growth of 50% of that of the drug-free control. Results were read after 24 h (yeasts) or 48 h (moulds) microdilution plates cultivation without agitation at t 35±2 °C in humidified atmosphere.

6.3. Results

Table S6. **In vitro antifungal activity screening. For abbreviations see section 4. Results expressed as MIC in µM read after 24 h (yeasts) or 48 h (filamentous fungi) of incubation.**

Compound	CA	CK	CP	CT	AF	AFla	LC	TI
PC-D-Ala	>500	>500	>500	>500	>500	>500	>500	>500
PC-L-Ala	>500	>500	>500	>500	>500	>500	>500	>500
PC-DL-Ala-Me	>500	250	>500	>500	>500	>500	>500	>500
PC-D-Ala-Et	>500	>500	>500	>500	>500	>500	>500	>500
PC-L-Ala-Et	>500	>500	>500	>500	>500	>500	>500	500
PC-D-Asp-diMe	NA	NA	NA	NA	NA	NA	NA	NA
PC-L-Asp-diEt	>500	>500	>500	>500	>500	>500	>500	>500
PC-L-*S*Bn-Cys	>250	>250	>250	>250	>250	>250	>250	>250
PC-D-Glu-diMe	>500	>500	>500	>500	>500	>500	>500	>500
PC-L-Glu-diEt	>500	>500	>500	>500	>500	>500	>500	>500
PC-Gly	>500	500	>500	>500	>500	>500	>500	>500
PC-Gly-Et	>500	>500	>500	>500	>500	>500	>500	>500
PC-D-Ile-Me	NA	NA	NA	NA	NA	NA	NA	NA
PC-L-Ile-Me	NA	NA	NA	NA	NA	NA	NA	NA
PC-DL-Leu-Et	>500	>500	>500	>500	>500	>500	>500	>500
PC-D-Leu-Me	>500	>500	>500	>500	>500	>500	>500	>500
PC-L-Leu-Me	>500	>500	>500	>500	>500	>500	>500	>500
PC-MeAcr	>125	>125	>125	>125	>125	>125	>125	>125
PC-L-Met-Me	>500	>500	>500	>500	>500	>500	>500	500
PC-D/L-Pgl-Me	>500	>500	>500	500	>500	>500	>500	>500
PC-DL-Phe-Et	>500	>500	>500	>500	>500	>500	>500	>500
PC-D-Ser	>500	>500	>500	>500	>500	>500	>500	500
PC-D-Ser-Me	>500	>500	>500	>500	>500	>500	>500	>500
PC-L-Ser-Me	>500	>500	>500	>500	>500	>500	>500	>500
PC-L-*O*Bn-Ser	>500	>500	>500	>500	>500	>500	>500	>500
PC-L-*O*Bn-Ser-Me	>250	>250	>250	125	>250	>250	>250	>250
PC-D-*Ot*Bu-Ser-Me	>500	>500	>500	>500	>500	>500	>500	>500
PC-L-*Ot*Bu-Ser-Me	>500	>500	>500	>500	>500	>500	>500	>500
PC-D-Thr-Me	>500	>500	>500	>500	>500	>500	>500	>500
PC-L-Thr-Me	>500	>500	>500	>500	>500	>500	>500	500
PC-D-*Ot*Bu-Thr-Me	>500	>500	>500	>500	>500	>500	>500	>500
PC-L-*Ot*Bu-Thr-Me	>500	>500	>500	>500	>500	>500	>500	>500
PC-D-Trp-Et	>500	>500	>500	>500	>500	>500	>500	>500
PC-L-Trp-Et	>500	>500	>500	>500	>500	>500	>500	>500
PC-D-Tyr-Me	>500	>500	>500	>500	>500	>500	>500	>500
PC-L-Tyr-Et	>500	>500	>500	>500	>500	>500	>500	>500
PC-DL-Val-Et	>500	>500	>500	>500	>500	>500	>500	>500
PC-D-Val-Me	>500	>500	>500	>500	>500	>500	>500	>500
PC-L-Val-Me	>500	>500	>500	>500	>500	>500	>500	>500

NA – results not available due to insolubility in the medium

7. Description of the supplied csv file

Table S7 Descriptors and properties contained in the csv file.

Descriptor	Class	Description
ast_fraglike	2D	Astex Fragment-like Test
ast_violation	2D	Astex Fragment-like Violation Count
a_acc	2D	Number of H-bond acceptor atoms
a_don	2D	Number of H-bond donor atoms
b_rotN	2D	Number of rotatable bonds
lip_acc	2D	Lipinski Acceptor Count
lip_don	2D	Lipinski Donor Count
lip_druglike	2D	Lipinski Druglike Test
lip_violation	2D	Lipinski Violation Count
logP(o/w)	2D	Log octanol/water partition coefficient
opr_leadlike	2D	Oprea Leadlike Test
opr_nring	2D	Oprea Ring Count
opr_nrot	2D	Oprea Rotatable Bond Count
opr_violation	2D	Oprea Violation Count
TPSA	2D	Topological Polar Surface Area (A^2)
Weight	2D	Molecular weight (CRC)
Relation		Equals to/higher than/lower than
Value		
Unit		
Type		

8. References

1. Franzblau, S.G.; Witzig, R.S.; McLaughlin, J.C.; Torres, P.; Madico, G.; Hernandez, A.; Degnan, M.T.; Cook, M.B.; Quenzer, V.K.; Ferguson, R.M., et al. Rapid, low-technology MIC determination with clinical Mycobacterium tuberculosis isolates by using the microplate Alamar Blue assay. *J Clin Microbiol* **1998**, *36*, 362-366.
2. European Committee for Antimicrobial Susceptibility Testing (EUCAST) of the European Society for Clinical Microbiology and Infectious Diseases (ESCMID). EUCAST Discussion Document E. Dis 5.1: determination of minimum inhibitory concentrations (MICs) of antibacterial agents by broth dilution. Clin Microbiol Infec 2003; 9:1-7. Availabe online: http://www.eucast.org/documents/publications_in_journals/ (accessed on 11 December, 2019).
3. EUCAST DEFINITIVE DOCUMENT E.DEF 7.3.1. Method for the determination of broth dilution minimum inhibitory concentrations of antifungal agents for yeasts. 2017. Availabe online: http://www.eucast.org/astoffungi/methodsinantifungalsusceptibilitytesting/susceptibility_testing_of_yeasts/ (accessed on 11 December, 2019).
4. EUCAST DEFINITIVE DOCUMENT E.DEF 9.3.1. Method for the determination of broth dilution minimum inhibitory concentrations of antifungal agents for conidia forming moulds. 2017. Availabe online: http://www.eucast.org/astoffungi/methodsinantifungalsusceptibilitytesting/susceptibility_testing_of_moulds/ (accessed on 11 December, 2019).
